# CircMIB2 therapy can effectively treat pathogenic infection by encoding a novel protein

**DOI:** 10.1038/s41419-023-06105-3

**Published:** 2023-08-31

**Authors:** Weiwei Zheng, Linchao Wang, Shang Geng, Liyuan Yang, Xing Lv, Shiying Xin, Tianjun Xu

**Affiliations:** 1 Laboratory of Fish Molecular Immunology, College of Fisheries and Life Science, Shanghai Ocean University, Shanghai, China; 2 Laboratory of Marine Biology and Biotechnology, Qingdao National Laboratory for Marine Science and Technology, Qingdao, China

**Keywords:** Immunosuppression, Long non-coding RNAs

## Abstract

The mRNA therapy is widely used in the treatment of diseases due to its efficient characteristics, and the COVID-19 vaccine is the application of mRNA therapy. However, due to the instability of mRNA, mRNA vaccines often need lots of modifications to ensure its stability. Recent research shows that circRNA with stable RNA structure can encode protein, which provides a new direction for mRNA therapy. Here, we discovered a novel circRNA (circMIB2) derived from E3 ubiquitin-protein ligase MIB2 (MIB2) gene in lower vertebrate fish, which can translate into a 134 amino acid protein (MIB2-134aa) through m^6^A modification, and is involved in innate immunity. MIB2-134aa is completely consistent with the amino acid sequence of the two domains of host gene MIB2 protein; host gene MIB2 can target TRAF6 through the two domains and inhibit the innate immune response by promoting the ubiquitination degradation of the K11-link of TRAF6, MIB2-134aa also targets TRAF6 through these same domains. Interestingly, MIB2-134aa greatly reduced the degradation of TRAF6 by its host gene MIB2. More importantly, we found that circRNA therapy of circMIB2 can significantly inhibit the colonization of *Vibrio anguillarum* in zebrafish, and it provides a new direction for the treatment of pathogenic diseases of fish.

## Introduction

RNA therapy is an effective method to treat or prevent diseases based on RNA molecules and mainly includes four strategies. The first strategy is that it can target and bind target nucleic acid molecules through single-stranded antisense RNA. For example, they can regulate the splicing of precursor mRNA and induce the degradation of target mRNA through degradation mediated by RNase H or block the translation of mRNA to protein [1, 2]. The second strategy is to combine target mRNA molecules with small interfering RNA (siRNA), and double-stranded siRNA drugs play their functions through cellular pathway RNAi to degrade target mRNA [3, 4]. The third strategy is RNA drugs targeting proteins, including RNA aptamers, which can bind and regulate the function of proteins [5]. The fourth strategy is RNA drugs that can be translated into proteins, and the most commonly used is modified mRNA [6]. After the modified mRNA is introduced into the cell, the mRNA is translated into protein and plays its role. Its great potential lies in its ability to carry out personalized immunotherapy in a relatively short time. Although the therapeutic effect of mRNA therapy is very strong, it has a big disadvantage in that its linear structure causes it to be very unstable and easy to degrade. Therefore, a lot of artificial modification of mRNA is required, which is not only time-consuming and laborious, but also may bring some by-products. Recent studies have shown that a more stable non-coding RNA molecule, circular RNA (circRNA), can also encode proteins, which undoubtedly provides a better choice for RNA therapy.

Different from ordinary linear mRNA or long non-coding RNA with 5’ m^7^G cap structure and 3’ poly (A) tail structure, circRNA is a covalently closed single-stranded RNA molecule [7–9]. It is precisely because of the covalently closed circular structure of circRNA that it has better stability than general linear mRNA, and its half-life is generally 18.8–23.7 h, while the half-life of general linear mRNA is only 4–7.4 h, which means that the degradation mechanism of circRNAs may be different from general linear mRNAs [7, 10]. The stable structure of circRNA is the most important basic characteristic that is expected to be used for RNA therapy. In recent years, circRNAs has also been found to encode functional small protein and play a certain function [11]. Such as the circSHPRH was found to encode a 146-amino acid protein, which protected full-length SHPRH from degradation by ubiquitin proteases [12]. Moreover, a novel tumor suppressor protein (AKT3-174aa) encoded by circular AKT3 RNA inhibits glioblastoma tumorigenicity [13]. In addition, FBXW7-185aa encoded by circFBXW7 could stabilize c-Myc and inhibit tumor cell proliferation and malignant phenotype [14]. The stable structure of circRNA and its ability to encode functional proteins are undoubtedly the status of circRNA as a substitute for linear mRNA in RNA therapy.

During the research, we found that many circRNA proteins have the exact same domains as their host gene proteins. However, no study has reported any specific connection between such circRNA proteins with host genes which has the same domain. Combined with the above report, it is not difficult to find that most circRNA proteins have strong functional links with their host gene proteins. CircRNA protein mainly affects host gene protein in two forms; one directly regulates their host gene protein functions by directly binding the host gene proteins, such as circLgr4-protein [15]; it can also indirectly regulate host gene protein stability or function by affecting other proteins such as SHPRH-146aa, AKT3-174aa, FBXW7-185aa, and β-catenin-370aa [12–14, 16]. Here, we want to know the significance of this type of circRNA protein which has the exact same domain as the host gene protein. Therefore, more relevant evidence is urgently needed to help understand the relationship between circRNA proteins and host gene proteins.

For insight into the relationship between circRNA proteins and their host gene proteins, a circRNA differentially expressed under SCRV virus infection conditions was selected. This circRNA encodes a protein that shares two identical domains with its host gene protein. We named this novel circRNA “circMIB2” derived from E3 ubiquitin-protein ligase MIB2 (MIB2) gene, which encodes a protein involved in innate immune in fish, miiuy croaker (*Miichthys miiuy*); in addition, circMIB2 mediates protein translation by modifying m^6^A. We found that the circRNA protein “MIB2-134aa” can compete with MIB2 for binding to TRAF6, thereby weakening the degradation of TRAF6 by MIB2 and promoting the innate immune response. This result provides definite evidence that circRNA proteins can indeed feedback regulate the function of its host gene protein by competitive mechanism through the same domain. More importantly, we found that circRNA therapy of circMIB2 can significantly improve the innate immune response of zebrafish and can significantly inhibit the colonization of *Vibrio anguillarum* in zebrafish. Compared with mRNA therapy, circRNA therapy has the advantages of low immunogenicity, high stability, and long duration of curative effect. Therefore, circRNA therapy is being extensively studied in human medicine, but it has not started in other species. Our research provides a reference for the first time for the application of circRNA therapy to the treatment of pathogenic diseases in economic fish.

## Results

### Characterization of circMIB2 involved in immunity

**Fig. 1:**
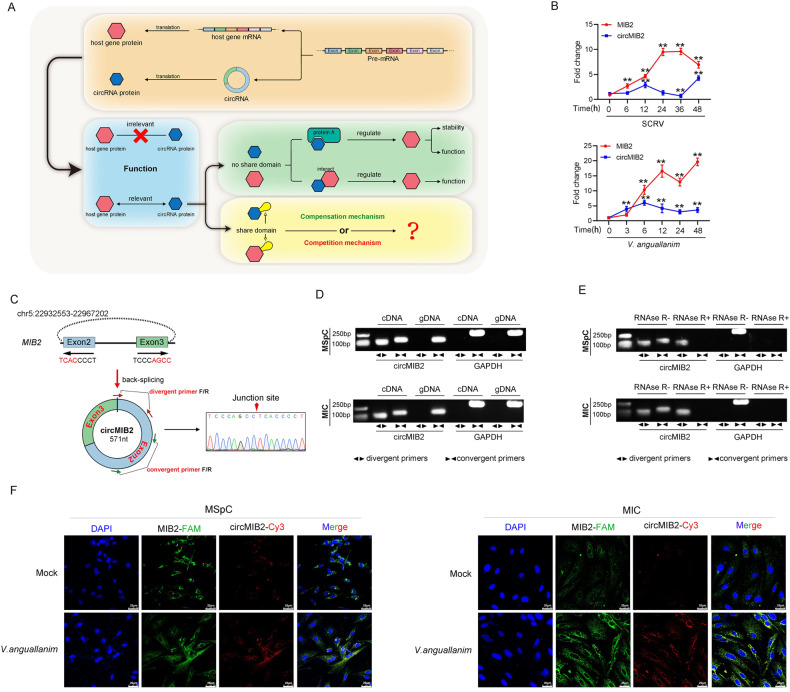
Characterization of circMIB2. **A** Schematic diagram of functions of circRNA-translated proteins. **B** The expression levels of MIB2 and circMIB2 in spleen samples were measured by qPCR at the indicated time after SCRV infection and *V. anguillarum* stimulation. **C** Confirmed the head-to-tail splicing of circMIB2 in the circMIB2 PCR product by Sanger sequencing. **D**, **E** The expression of circMIB2 and linear MIB2 mRNA in both MSpC and MIC cell lines was detected by PCR assay followed by nucleic acid electrophoresis assay in the presence or absence of RNase R. **F** Detect the distribution and proportion of MIB2 mRNA and circMIB2 in MIC and MSpC cells through RNA FISH assay. All data represented the three independent triplicated experiments. **p < 0.01.

Whether the abovementioned circRNA proteins with the same domain as the host gene protein have a functional connection with the host gene protein is still unknown (Fig. 1A), and we carried out the following research by using the representative fish of lower vertebrates as a model. First, we treated miiuy croaker with SCRV virus, then performed RNA-seq analysis to compare the circRNA expression levels between SCRV-treated and untreated spleen (GenBank accession no. PRJNA685924). We found a circular RNA (circMIB2) was differentially expressed and has coding potential.

At the same time, we also detected the expression of the host gene of circMIB2 and MIB2 after SCRV and *Vibrio anguillarum* infection. The results showed that circMIB2 and MIB2 were all significantly up-regulated in miiuy croaker spleen tissue treated with SCRV and *V. anguillarum* at different time points (Fig. 1B). The transcriptome sequencing revealed that circMIB2 was 571 nt in length (Fig. 1C). Using miiuy croaker whole-genome to perform blast analysis on the host gene MIB2, it was found that MIB2 gene was located on chromosome 5, and circMIB2 was back-splicing only exon2 and exon3, without any intron in the middle. First, circMIB2 divergent primers were designed for PCR amplification, and the amplified products were subjected to Sanger sequencing to confirm that circMIB2 was spliced from the head to the tail (Fig. 1C). Then, we used convergent primers to amplify MIB2 gene and divergent primers to amplify circMIB2, we designed the convergent primer on the exon 2 of MIB2 and design divergent primers at the junction site of circMIB2 (Fig. 1C). cDNA and gDNA were extracted separately from *M. miiuy* spleen cells (MSpC) and *M. miiuy* intestine cells (MIC) and subjected to PCR and agarose gel electrophoresis assays. The results shown in Fig. 1D indicated that circMIB2 was amplified from cDNA by using only divergent primers, whereas no amplification product was observed from gDNA. Considering that stability was a crucial characteristic of circRNAs, we thus employed RNase R to confirm the stability of circMIB2. The results from the agarose gel electrophoresis assay showed that circMIB2, rather than linear MIB2 or GAPDH, resisted digestion by RNase R (Fig. 1E). We then evaluated the expression levels of circMIB2 in *M. miiuy* kidney cells (MKC), MSpC, MIC, *M. miiuy* brain cells (MBrC), *M. miiuy* muscle cells (MMC), and *M. miiuy* liver cells (MLC), and these cell lines were divided into two groups, one treated with RNase R and the other untreated (Supplementary Fig. 1A). Among the aforementioned cell lines, MSpC and MIC cells showed the highest and lowest the expression of circMIB2, respectively. The mRNA expression of MIB2 decreased significantly after RNase R treatment in all aforementioned cell lines, while the expression of circMIB2 almost did not decrease in all aforementioned cell lines (Supplementary Fig. 1A). Further detection of MIB2 mRNA and circMIB2 in MIC and MSpC cells were conducted through RNA FISH assay. The MIB2 mRNA and circMIB2 were mainly distributed in the cytoplasm of MIC and MSpC cells, and under normal circumstances, circMIB2 was very low. However, the content of circMIB2 significantly increased after *V. anguillarum* infection (Fig. 1F). Therefore, we selected MSpC and MIC to investigate the function and regulatory mechanism of circMIB2.

In addition, we detected the distribution of circMIB2 by cytoplasmic nuclear fractionation experiments and found that circMIB2 was primarily localized in the cytoplasm (Supplementary Fig. 1B). Accordingly, these results suggested that circMIB2 was a stable circRNA expressed and primarily distributed in the cytoplasm.

### circMIB2 encodes a 134 amino acid (aa) novel protein, MIB2-134aa

**Fig. 2:**
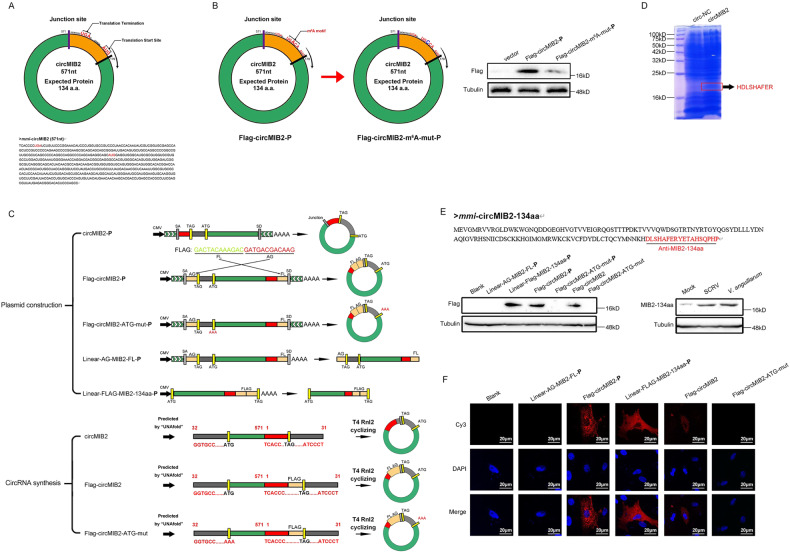
circMIB2 encodes a 134 amino acid (aa) novel protein, MIB2-134aa. **A** The putative ORF in circMIB2. Lower panel, the sequences of the putative ORF are shown. **B** Schematic diagram of Flag-circMIB2-m^6^A-mut plasmid construction. MSpC cells were transfected with Flag-circMIB2-P or Flag-circMIB2-m^6^A-mut-P plasmids and detected by WB. **C** Upper panel, the schematic diagram of circMIB2-P, Flag-circMIB2-P, Flag-circMIB2-ATG-mut-P, Linear-AG-MIB2-FL-P, and Linear-FLAG-MIB2-134aa-P plasmid construction. Lower panel, the schematic diagram of the synthesis of circRNA circMIB2, Flag-circMIB2, Flag-circMIB2-ATG-mut in vitro. **D** Total proteins from circ-NC and circMIB2-transfected MIC cells were separated via SDS-PAGE. The differential gel bands were extracted and subjected to LC-MS. **E** Upper panel, the putative MIB2-134aa amino acid sequences and antibody generation region was shown as indicated to produce the MIB2-134aa antibody. Lower: Flag tag antibody was used to detect MIB2-134aa expression in MIC cells transfected with the plasmids and circRNAs mentioned in (**C**). MIB2-134aa antibody was used to detect MIB2-134aa expression in MSpC cells after SCRV and *V. anguillarum* infection. **E** Linear-AG-MIB2-FL-P, Flag-circMIB2-P, Linear-Flag-MIB2-134aa-P, Flag-circMIB2, and Flag-circMIB2-ATG-mut were transfected into MSpC cells. Original magnification is 400; All data represent the means ± SE from three independent triplicate experiments.

circMIB2 has a predicted ORF, which may encode a 134 aa protein by using an overlapping start-stop codon “UGA……AUG” (Fig. 2A). Through prediction, we found that no IRES sequence exists on circMIB2 but a m^6^A modification site. Therefore, we constructed the potential m^6^A site mutant plasmids of circMIB2 (Fig. 2B). Then, we transfected the wild-type and m^6^A site mutant FLAG-circMIB2-P plasmids into MSpC cells, respectively. The results showed that the MIB2-134aa protein expressed by the wild-type FLAG-circMIB2-P plasmid was significantly higher than that of the mutant-type plasmid. This result suggests that m^6^A modification may indeed exist on circMIB2. To construct circMIB2 plasmid (circMIB2-P) and Flag-circMIB2 plasmid (Flag-circMIB2-P), the full-length circMIB2 sequence or full-length circMIB2 sequence with Flag sequence was amplified by specific primer pairs and cloned into pLC5-circ vector. Construction of initial codon mutant of Flag-circMIB2-P plasmid (Flag-circMIB2-ATG-mut-P), and based on Flag-circMIB2-P plasmid, the initial codon “ATG” was mutated to “AAA”. To construct the Linear-AG-MIB2-FL-P and Linear-Flag-MIB2-134aa-P plasmids (Fig. 2C). To further verify that circMIB2 can encode proteins, we use T4 RNA Ligase 2 (T4 Rnl2) to synthesize circRNA circMIB2 in vitro. In addition, we also synthesized circMIB2 (Flag-circMIB2) with Flag tag and the initial codon mutant of circMIB2 (Flag-circMIB2-ATG-mut), and we synthesized circ-NC as the control of circMIB2 (Fig. 2C). We next used liquid chromatography Tandem Mass Spectrometer (LC-MS) to characterize the amino acid sequences of MIB2-134aa in circRNA circMIB2-transfected MIC cells. As MIB2-134aa is formed by the “spanning junction ORF,” the identified distinctive amino acids in this region further suggested that this novel protein was encoded by circMIB2 (Fig. 2D). We pulldown the endogenous MIB2-134aa protein with its antibody, followed by LC-MS, and the results showed that MIB2-134aa peptide could be detected (Supplementary Fig. 2B). We transfected the above-constructed plasmids and circRNAs into MIC cells respectively, and detected the expression of MIB2-134aa protein by Western Blotting (WB) and Immunological Fluorescence (IF) 48 h later. The results showed that the expression of MIB2-134aa protein could be detected in the samples transfected with Linear-Flag-MIB2-134aa-P and Flag-circMIB2-P plasmids, while the expression of MIB2-134aa protein could not be detected in the samples transfected with Flag-circMIB2-ATG-mut-P (left panel of Fig. 2E), In addition, the expression of MIB2-134aa protein can also be detected in the samples transfected with circRNA Flag-circMIB2, while the expression of MIB2-134aa protein can’t be detected in the samples transfected with circRNA Flag-circMIB2-ATG-mut (left panel of Fig. 2E). The above results further prove that circMIB2 can encode MIB2-134aa protein. The result of IF was consistent with that of WB, and the result of IF showed that MIB2-134aa protein was mainly distributed in the cytoplasm (Fig. 2F). In addition, we synthesized specific antibodies against MIB2-134aa protein according to the amino acid sequence of circMIB2 trans-cyclization site, and found that the expression of MIB2-134aa protein in MSpC cells was very low under normal conditions, but the expression of MIB2-134aa protein would significantly increase under the conditions of SCRV or *V. anguillarum* infection (right panel of Fig. 2E). In order to further test the specificity of the MIB2-134aa antibody, we conducted further validation by transfecting the circMIB2-P recombinant expression plasmid and si-circMIB2-1 into cells. The experimental results showed that MIB2-134aa could recognize the protein produced by the circMIB2-P recombinant expression plasmid and also recognize the endogenous MIB2-134aa protein (Supplementary Fig. 2A). However, due to the low expression level of MIB2-134aa protein under normal conditions, it is difficult to detect it, Therefore, to test the efficacy of si-circMIB2-1, we co-transfected the circMIB2-P plasmid to increase the background level of MIB2-134aa protein (Supplementary Fig. 2A). These results show that circMIB2 encodes MIB2-134aa protein, and this protein seems to play a very important role in the process of infection of the host by SCRV and *V. anguillarum*.

### circMIB2 and MIB2-134aa promote host innate immunity

**Fig. 3:**
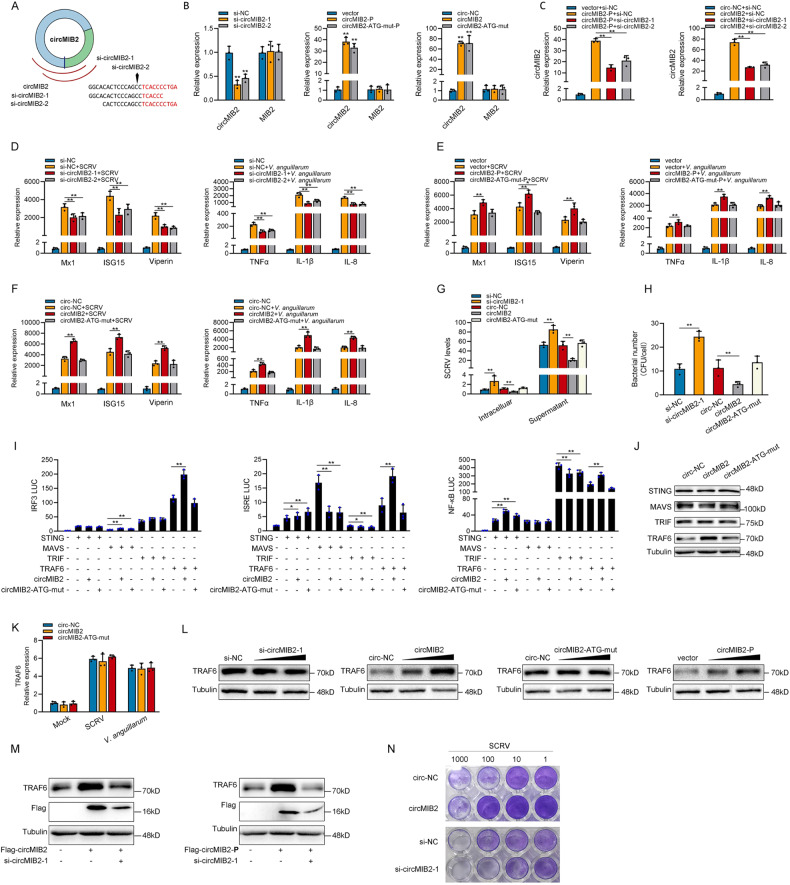
MIB2-134aa promotes host innate immunity. **A** The schematic diagram of siRNAs. **B** qPCR analysis of circMIB2 and linear MIB2 mRNA in MSpC cells treated with siRNAs, or in MIC cells overexpressing circMIB2-P and circMIB2-ATG-mut-P plasmids, or in MIC cells treated with circRNAs circMIB2 and circMIB2-ATG-mut. **C** qPCR analysis of circMIB2 and linear MIB2 mRNA in MSpC cells co-transfected with siRNAs and circMIB2-P plasmid or co-transfected with siRNAs and circMIB2. **D**–**F** qPCR assays were performed to determine the expression levels of antiviral genes and inflammatory factors in MSpC cells transfected with NC or siRNAs of circMIB2 (**D**) and in MIC cells transfected with vector or circMIB2-P or circMIB2-ATG-mut-P plasmids (**E**), and in MIC cells transfected with circ-NC or circMIB2 or circMIB2-ATG-mut after SCRV or *V. anguillarum* infection (**F**). **G** The qPCR analysis was conducted for intracellular and supernatant SCRV RNA expression in MSpC cells transfected with si-NC or si-circMIB2-1 and circ-NC or circMIB2 or circMIB2-ATG-mut infected with SCRV. **H** MSpC cells were transfected with si-NC or si-circMIB2 and circ-NC or circMIB2-1 or circMIB2-ATG-mut and then infected with *V. anguillarum*. **I** Relative luciferase activities were detected in MSpC after co-transfection with STING, MAVS, TRIF, and TRAF6 expression plasmid, phRL-TK *Renilla* luciferase plasmid, luciferase reporters, circMIB2 or circMIB2-ATG-mut. **J** WB detection of STING, MAVS, TRIF, and TRAF6 protein level after circ-NC or circMIB2 or circMIB2-ATG-mut transfection. **K** qPCR analysis of TRAF6 mRNA in MIC cells transfected with circ-NC or circMIB2 or circMIB2-ATG-mut. **L** Protein levels of TRAF6 in MSpC cells after transfected with si-NC or si-circMIB2-1, and in MIC cells after transfected with circ-NC or circMIB2 or circMIB2-ATG-mut and transfected with vector or circMIB2-P plasmid. **M** Protein levels of TRAF6 in MSpC cells after co-transfected with Flag-circMIB2 and si-circMIB2-1 or co-transfected with Flag-circMIB2-P and si-circMIB2-1. **N** MSpC and MIC cells seeded in 48-well plates overnight were treated with cultural supernatants at the dose indicated for 48 h. Then, cell monolayers were fixed with 4% paraformaldehyde and stained with 1% crystal violet. MSpC cells were transfected with si-NC or si-circMIB2; MIC cells were transfected with circ-NC or circMIB2. All data represented the mean ± SE from three independent triplicated experiments. *p < 0.05; **p < 0.01.

We designed the two small interfering RNAs (siRNA) against circMIB2 (Fig. 3A). Consequently, two siRNAs (si-circMIB2-1 and si-circMIB2-2) evidently decreased the circMIB2 expression level, but such siRNAs did not affect the expression level of linear MIB2 mRNA in MSpC. As si-circMIB2-1 could induce higher inhibitory efficiency (left panel of Fig. 3B). The circMIB2-P and circMIB2-ATG-mut-P plasmid could significantly increase the circMIB2 expression levels rather than linear MIB2 mRNA in MIC cells (middle panel of Fig. 3B). Moreover, the circMIB2 and circMIB2-ATG-mut plasmid could significantly increase the circMIB2 expression levels rather than linear MIB2 mRNA in MIC cells, and the effect of circMIB2 to increase the level of circMIB2 RNA is better than that of circMIB2-P plasmid (right panel of Fig. 3B). We co-transfected siRNAs of circMIB2 with circMIB2-P plasmid or circMIB2 into MSpC cells respectively. The results showed that siRNAs of circMIB2 could significantly inhibit the increase of circMIB2 expression caused by circMIB2-P plasmid or circRNA circMIB2 (Fig. 3C). As shown in Fig. 3D, silence of circMIB2 decreased the expression levels of these genes under SCRV infection or *V. anguillarum* infection. By contrast, the overexpression of circMIB2-P plasmid (Fig. 3E) or circRNA circMIB2 (Fig. 3F) could significantly increase these genes under SCRV infection or *V. anguillarum* infection. Moreover, the overexpression of circMIB2-ATG-mut-P plasmid (Fig. 3E) or circRNA circMIB2-ATG-mut (Fig. 3F) both has no effect on these genes under SCRV infection or *V. anguillarum* infection. Additionally, we assessed the impact of circMIB2 on ISG15, TNFα, and IL-8 protein levels. Our experimental findings were consistent with qPCR experiments (Supplementary Fig. 2C). Overexpressing circMIB2 significantly boosted ISG15 protein levels while knocking it down inhibited ISG15 protein expression during SCRV infection. Moreover, overexpressing circMIB2 substantially raised TNFα and IL-8 protein levels, while knocking it down significantly reduced TNFα and IL-8 protein expression during *V. anguillarum* infection. The above results show that circMIB2 can participate in the innate immune response caused by SCRV or *V. anguillarum*.

Furthermore, we examined the effect of circMIB2 on SCRV replication to explore the biological significance of circMIB2 in SCRV-induced host cells. We found that silence of circMIB2 significantly promoted SCRV replication (Fig. 3G). And circMIB2 could significantly inhibit SCRV replication but not circMIB2-ATG-mut (Fig. 3G). To further confirm the effect of circMIB2 on *V. anguillarum* invading cells, the plate counting method was used. The results showed that silence of circMIB2 could significantly increase the *V. anguillarum* invading MIC cells (Fig. 3H). And circMIB2 could significantly inhibit the *V. anguillarum* invading MIC cells but not circMIB2-ATG-mut (Fig. 3H). We wanted to know which gene both in the antiviral antibacterial signal pathway of teleost is affected by MIB2-134aa, so we tested STING, MAVS, TRIF, and TRAF6 through the dual-luciferase reporter system. Then, the STING, MAVS, TRIF, and TRAF6 plasmid with only CDS; circMIB2; circMIB2-ATG-mut; and various reporter gene plasmids were co-transfected into EPC cells. Luciferase readouts here represent the transcriptional activation degree of these reporter genes. Co-transfection of these immune-related proteins can activate the reporter genes. The activation degree of the reporter genes has changed after transfection of the wild-type or ATG mutant of circMIB2, indicating that circMIB2 may be involved in the transmission of this signal pathway. If the wild type of circMIB2 has a significant regulatory trend for the reporter gene activated by an immune-related protein, but its ATG mutant has no regulatory trend, it indicates that the MIB2-134aa is playing a role rather than circMIB2 itself. The results showed that MIB2-134aa promoted the activity of reporter genes such as IRF3, ISRE, and NF-κB mainly by affecting the activity of TRAF6 (Fig. 3I). In order to further verify the correctness of the results of the double luciferase experiment, we detected the effect of circMIB2 on protein by WB in MIC cells. The result shows that circMIB2 could only significantly increase the protein levels of TRAF6 but not STING, MAVS, and TRIF, and circMIB2-ATG-mut has no effect on TRAF6 (Fig. 3J). We tested the effect of circMIB2 and circMIB2-ATG-mut on TRAF6 mRNA, and the results showed that circMIB2 and circMIB2-ATG-mut did not affect the level of TRAF6 mRNA (Fig. 3K). As shown in Fig. 3L, the si-circMIB2-1 could significantly inhibit the level of TRAF6 protein, and circMIB2 and circMIB2-P could significantly promote the expression of TRAF6 protein, while circMIB2-ATG-mut had no effect on TRAF6 protein. As shown in Fig. 3M, the si-circMIB2-1 could reverse the promotion effect of circMIB2 or circMIB2-P on TRAF6. As shown in Fig. 3N, after transfection with circMIB2, the CPE (cytopathic effect) was significantly reduced under SCRV infection conditions, while knocking down circMIB2 resulted in a significant increase in CPE. Collectively, MIB2-134aa could promote TRAF6-mediated innate immune response by targeting TRAF6 protein; all the experimental results show that the promotion of circMIB2 on innate immune response mainly comes from the role of its encoded protein MIB2-134aa.

### Host gene MIB2 inhibits host innate immunity

**Fig. 4:**
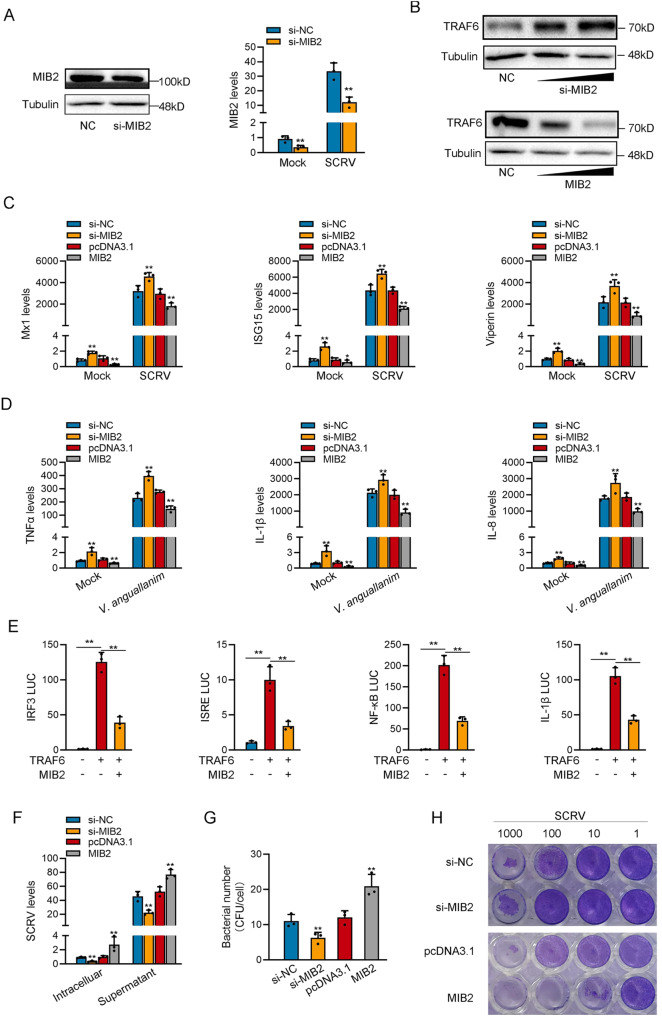
MIB2 inhibits host innate immunity. **A** Relative protein and mRNA levels of MIB2 in MSpC cells after transfected with si-NC or si-circMIB2. **B** Relative protein levels of TRAF6 in MSpC cells after transfected with si-NC or si-MIB2 and in MIC cells with pcDNA3.1 or MIB2. **C**, **D** qPCR assays were performed to determine the expression levels of antiviral genes in MSpC cells transfected with si-NC or si-MIB2 and vector or MIB2 after SCRV infection (**C**) and determine the expression levels of T inflammatory factors in MIC cells transfected with si-NC or si-MIB2 and pcDNA3.1 or MIB2 after *V. anguillarum* infection (**D**). **E** Relative luciferase activities were detected in MSpC after co-transfection with TRAF6 expression plasmid, phRL-TK *Renilla* luciferase plasmid, luciferase reporters, pcDNA3.1, MIB2. **F** MSpC cells transfected with NC or si-MIB2 and pcDNA3.1 or MIB2, respectively, then infected with SCRV. The qPCR analysis was conducted for SCRV RNA expression. **G** MIC cells were transfected with NC or si-MIB2 and pcDNA3.1 or MIB2 and then infected with *V. anguillarum*. **H** MSpC and MIC cells seeded in 48-well plates overnight were treated with cultural supernatants at the dose indicated for 48 h. Then, cell monolayers were fixed with 4% paraformaldehyde and stained with 1% crystal violet. MSpC cells were transfected with si-NC or si-MIB2; MIC cells were transfected with vector or MIB2. All data represented the mean ± SE from three independent triplicated experiments. *p < 0.05; **p < 0.01.

MIB2-134aa and MIB2 proteins have two same domains, so we believe that there is a certain connection between the two proteins. We designed the siRNA against MIB2, and the plasmid of MIB2 was constructed to explore the biological function of MIB2. Consequently, the siRNA (si-MIB2) evidently decreased the MIB2 protein level and mRNA level in MSpC cells (Fig. 4A). We detected whether MIB2 could affect the expression level of TRAF6 protein. The result shows that silence of MIB2 significantly increased the protein levels of TRAF6, and overexpression of MIB2 significantly decreased the protein levels of TRAF6 (Fig. 4B). As shown in Fig. 4C, D, silence of MIB2 increased the expression levels of these genes under SCRV or *V. anguillarum* infection. By contrast, the overexpression of MIB2 could significantly inhibit the expression levels of these genes after SCRV or *V. anguillarum* infection. Additionally, we assessed the impact of MIB2 on ISG15, TNFα, and IL-8 protein levels. Our experimental findings were consistent with qPCR experiments (Supplementary Fig. 2D). Overexpressing MIB2 significantly inhibited ISG15 protein levels, while knocking it boosted ISG15 protein expression during SCRV infection. Moreover, overexpressing MIB2 substantially reduced TNFα and IL-8 protein levels, while knocking it down significantly raised TNFα and IL-8 protein expression during *V. anguillarum* infection. Then, the TRAF6 plasmid with only CDS, MIB2 plasmid, and various reporter gene plasmids were co-transfected into EPC cells. The results showed that MIB2 inhibited the activity of reporter genes such as IRF3, ISRE, NF-κB, and IL-1β mainly by affecting the expression of TRAF6 (Fig. 4E).

Furthermore, we found that silence of MIB2 significantly inhibited SCRV replication after SCRV infection, and MIB2 overexpression significantly promoted SCRV replication (Fig. 4F). In addition, knockdown of MIB2 could significantly decrease the *V. anguillarum* invading MIC cells (Fig. 4G). As shown in Fig. 4H, after knocking down MIB2, the CPE (cytopathic effect) was significantly reduced under SCRV infection conditions, while transfection with circMIB2 resulted in a significant increase in CPE. Collectively, MIB2 could inhibit TRAF6-mediated innate immune response by promoting the degradation of TRAF6 protein, and all results suggest that MIB2 has the opposite function to MIB2-134aa.

### MIB2-134aa competes with MIB2 for binding TRAF6 to regulate the ubiquitination

**Fig. 5:**
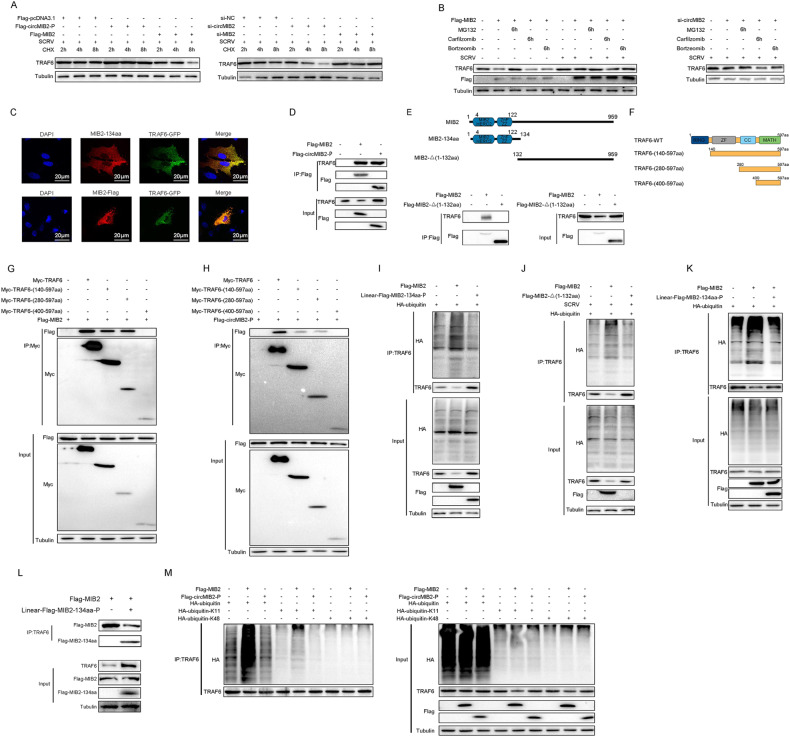
MIB2-134aa competes with MIB2 for binding TRAF6 to regulate the ubiquitination. **A** MSpC and MIC cells were transfected with Flag-MIB2 or Flag-circMIB2-P plasmids or silence MIB2 or circMIB2, respectively, and treated with 10 μM CHX. **B** MSpC cells were transfected with Flag-MIB2 or si-circMIB2 and treated with DMSO or 10 μM MG132 or 10 μM Carfilzomib or 10 μM Bortzeomib. **C** Flag-MIB2 was co-transfected with GFP-TRAF6 into MSpC cells. Original magnification is 400. **D** Immunoprecipitation and immunoblot analysis of Flag-MIB2 or Linear-Flag-MIB2-134aa-P in MSpC cells. **E** Upper panel: Schematic diagram of MIB2-Δ(1-132aa) plasmid construction. Lower panel: Immunoprecipitation and immunoblot analysis of Flag-MIB2, Flag-MIB2-Δ(1-132aa) or Linear-Flag-MIB2-134aa-P, in MSpC cells. **F** Schematic diagram of TRAF6 and TRAF6 domain mutant plasmid construction. **G** Immunoprecipitation and immunoblot analysis of Flag-MIB2, with TRAF6 and TRAF6 mutant in MSpC cells. **H** Immunoprecipitation and immunoblot analysis of Linear-Flag-MIB2-134aa-P with TRAF6 and TRAF6 mutant in MSpC cells. **I** Coimmunoprecipitation analysis of TRAF6 ubiquitination in MSpC cells transfected HA-ubiquitin-WT in the presence of control vector, Flag-MIB2 or Linear-Flag-MIB2-134aa-P expression plasmid. **J** Coimmunoprecipitation analysis of TRAF6 ubiquitination in MSpC cells transfected HA-ubiquitin-WT in the presence of control vector, Flag-MIB2, or Flag-MIB2-Δ(1-132aa), or Linear-Flag-MIB2-134aa-P expression plasmid. **K** Coimmunoprecipitation analysis of TRAF6 ubiquitination in MSpC cells transfected HA-ubiquitin-WT in the presence of control vector, Flag-MIB2, or Flag-MIB2 and Linear-Flag-MIB2-134aa-P expression plasmid. **L** Immunoprecipitation and immunoblot analysis of Flag-MIB2 or Linear-Flag-MIB2-134aa-P, in MSpC cells. **M** Coimmunoprecipitation analysis of TRAF6 ubiquitination in MSpC co-transfected with Flag-MIB2 or Linear-Flag-MIB2-134aa-P expression plasmid and HA-ubiquitin-WT, HA-ubiquitin-K11 or HA-ubiqutin-K48 plasmids. All data represented the three independent triplicated experiments.

To determine which way was used to regulate TRAF6 degradation by MIB2-134aa and MIB2. Therefore, we first explored the effects of circMIB2 and MIB2 on the stability of TRAF6 protein. As shown in Fig. 5A, the results showed that overexpression of MIB2 could weaken the stability of TRAF6 protein, while silence of MIB2 could increase the stability of TRAF6 protein, and MIB2-134aa has the exact opposite effect. To further explore how TRAF6 protein is degraded, we tried to use protease inhibitors to block the degradation of circMIB2 and MIB2 on TRAF6. The results are shown in Fig. 5B, the protease inhibitor MG132 can effectively prevent MIB2 from degrading TRAF6 protein. We found that MG132 can effectively prevent TRAF6 degradation caused by silencing circMIB2. These results indicated that MIB2-134aa or MIB2 may be able to regulate TRAF6 degradation through the proteasome pathway. Immunostaining with specific antibodies showed a very high degree of colocalization both between MIB2-134aa and GFP-TRAF6 or Flag-MIB2 and GFP-TRAF6 transfected into MSpC cells (Fig. 5C).

To confirm that MIB2-134aa and MIB2 target TRAF6, we investigated the interaction between MIB2-134aa and TRAF6 or MIB2 and TRAF6. When co-transfected into MSpC cells, Flag-MIB2 coimmunoprecipitated with TRAF6, and the Flag-circMIB2-P also coimmunoprecipitated with TRAF6 (Fig. 5D). Because MIB2 and MIB2-134aa share two same domains, we want to know whether this two domains plays a role in binding to TRAF6. Therefore, we constructed the domain deletion mutant of MIB2 (Flag-MIB2-Δ(1-132aa)) plasmid. Immunocoprecipitation assay showed that the binding ability of TRAF6 to MIB2 decreased significantly after the domain was deletion (Fig. 5E).

To further explore which region of TRAF6 MIB2-134aa and MIB2 mainly bind to, we constructed the TRAF6 deletion mutant plasmid (Fig. 5F). Immunocoprecipitation assay showed that MIB2 mainly binds to the region between amino acids 280 and 400 of TRAF6 protein (Fig. 5G), and MIB2-134aa also mainly binds to the region between amino acids 280 and 400 of TRAF6 protein (Fig. 5H). Coimmunoprecipitation experiments showed that TRAF6 ubiquitination was markedly increased in the presence of MIB2 but not MIB2-134aa expression plasmid (Fig. 5I). Then we tested whether MIB2 without the two domains could continue to promote the ubiquitination modification of TRAF6. The results showed that MIB2-Δ(1-132aa) could also not promote the ubiquitination of TRAF6 (Fig. 5J). We also found that MIB2-134aa was able to reverse MIB2-mediated ubiquitination of TRAF6 (Fig. 5K). When we co-transfected the Flag-MIB2 and Linear-MIB2-134aa-P plasmids into cells, we found that the interaction effect between TRAF6 and MIB2 was significantly weakened. Therefore, we believe that MIB2-134aa and MIB2 can compete to bind to TRAF6 protein (Fig. 5L). To further investigate MIB2 and MIB2-134aa-mediated TRAF6 ubiquitination, we used mutants in which all lysine residues except K11 or K48 were replaced with arginine (HA-ubiquitin-K11 and HA-ubiquitin-K48). MIB2-mediated ubiquitination of TRAF6 was significantly increased in the presence of wild-type HA-ubiquitin (HA-ubiquitin-WT) or HA-ubiquitin-K11 (Fig. 5M). These data indicate that MIB2 mediates K11-linked ubiquitination, and the two domains are the key to the interaction between MIB2 and TRAF6.

### N6-methyladenosine (m6A) modification mediates circMIB2 translation proteins

**Fig. 6:**
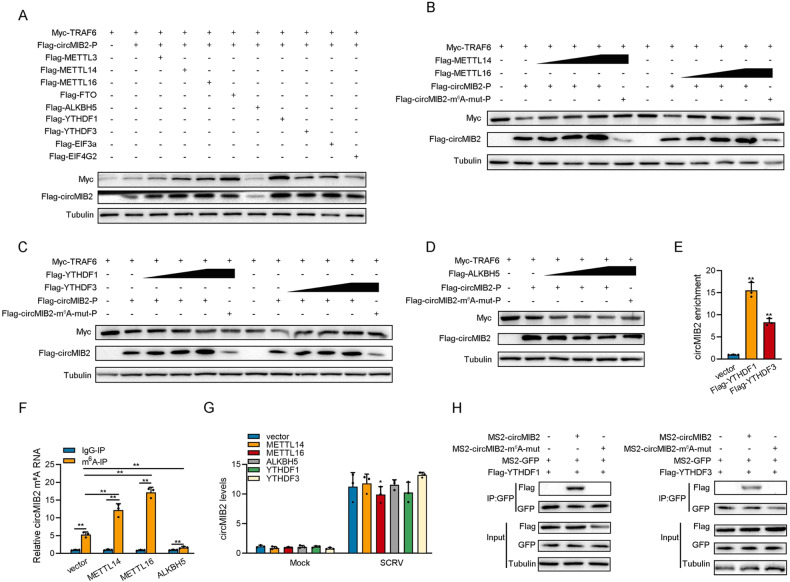
N6-methyladenosine modification mediates circMIB2 translation proteins. **A** Myc-TRAF6 and Flag-circMIB2-P were co-transfected into MSpC cells with m^6^A modification-related genes, respectively, and then the protein levels of Myc-TRAF6 and Flag-circMIB2 were detected. **B**–**D** Myc-TRAF6 and Flag-circMIB2-P were co-transfected into MSpC cells with METTL14 or METTL16 respectively (**B**), Myc-TRAF6 and Flag-circMIB2-P were co-transfected into MSpC cells with YTHDF1 and YTHDF3 respectively (**C**), Myc-TRAF6 and Flag-circMIB2-P were co-transfected into MSpC cells with ALKBH5 (**D**), and then the protein levels of Myc-TRAF6 and Flag-circMIB2 were detected. **E** The level of circMIB2 upon YTHDF1 or YTHDF3 overexpression was examined by RIP-qPCR. **F** The m^6^A level alteration of circMIB2 upon METTL14, METTL16, or ALKBH5 overexpression were examined by MeRIP-qPCR. **G** Relative RNA levels of circMIB2 in MSpC cells after transfected with pcDNA3.1, METTL14, METTL16, YTHDF1, YTHDF3, and ALKBH5, respectively. **H** The protein level of YTHDF1 or YTHDF3 was examined by RNA pulldown. All data represented the mean ± SE from three independent triplicated experiments. *p < 0.05; **p < 0.01.

Through prediction, we found that no IRES sequence exists on circMIB2 but an m^6^A modification site. Therefore, we constructed the potential m^6^A site mutant plasmids of circMIB2 (Fig. 2B). Then, we constructed some plasmids of m^6^A modification-related genes. Subsequently, co-transfection experiments were conducted to verify whether these m^6^A modification-related genes could affect the translation efficiency of circMIB2. As shown in Fig. 6A, the results indicated that METTL14, METTL16, YTHDF1, and YTHDF3 could significantly increase the expression of MIB2-134aa protein and significantly increase the protein level of TRAF6. It was also found that ALKBH5 could significantly reduce the expression of MIB2-134aa protein and significantly reduce the protein level of TRAF6 (Fig. 6A). In order to verify the above experimental results, we conducted a concentration gradient experiment. The experimental results showed that METTL14, METTL16, YHTDF1, and YTHDF3 could significantly promote the expression of proteins in wild-type Flag-circMIB2-P plasmids but could not promote the expression of proteins in m^6^A site mutant plasmids (Fig. 6B, C). And results showed that ALKBH5 could significantly induce the expression of proteins in wild-type Flag-circMIB2-P plasmids but could not induce the expression of proteins in m^6^A site mutant plasmids (Fig. 6D).

The expression of circMIB2-134aa proteins was closely related to the level of TRAF6 protein; when the expression of circMIB2-134aa proteins increased, TRAF6 would correspondingly increase, showing a significant positive correlation, while ALKBH5 could significantly inhibit the expression of protein in wild-type circMIB2-P plasmid. In order to verify whether YTHDF1, YTHDF3 and MIB2 can directly interact with circMIB2, we conducted relevant RIP experiments. The experimental results showed that YTHDF1 and YTHDF3 can significantly enrich circMIB2, of which YTHDF1 and YTHDF3 both have a high enrichment efficiency (Fig. 6E). Furthermore, we explored its effect on the methylation level of circMIB2 by performing a MeRIP-qPCR assay. Our results showed that overexpression of METTL14 and METTL16 dramatically increased the m^6^A level of circMIB2 mRNA (Fig. 6F); in contrast, the m^6^A levels of circMIB2 were significantly decreased after overexpression of ALKBH5 (Fig. 6F). Then we tested the effects of METTL14, METTL16, ALKBH5, YTHDF1, and YTHDF3 on the RNA level of circMIB2. The results showed that METTL14, METTL16, ALKBH5, YTHDF1, and YTHDF3 had no significant effect on the RNA level of circMIB2 (Fig. 6G). At the same time, we also conducted RNA pulldown experiments. The results showed that circMIB2 could enrich YTHDF1, YTHDF3 proteins, while m^6^A site mutant circMIB2 could not enrich YTHDF1, YTHDF3 proteins (Fig. 6H). Based on the above results, we believe that circMIB2 can mediate the translation of proteins through m^6^A modification.

### CircRNA therapy of circMIB2 promotes the innate immune response of zebrafish and inhibits the colonization of *V. anguillarum*

**Fig. 7:**
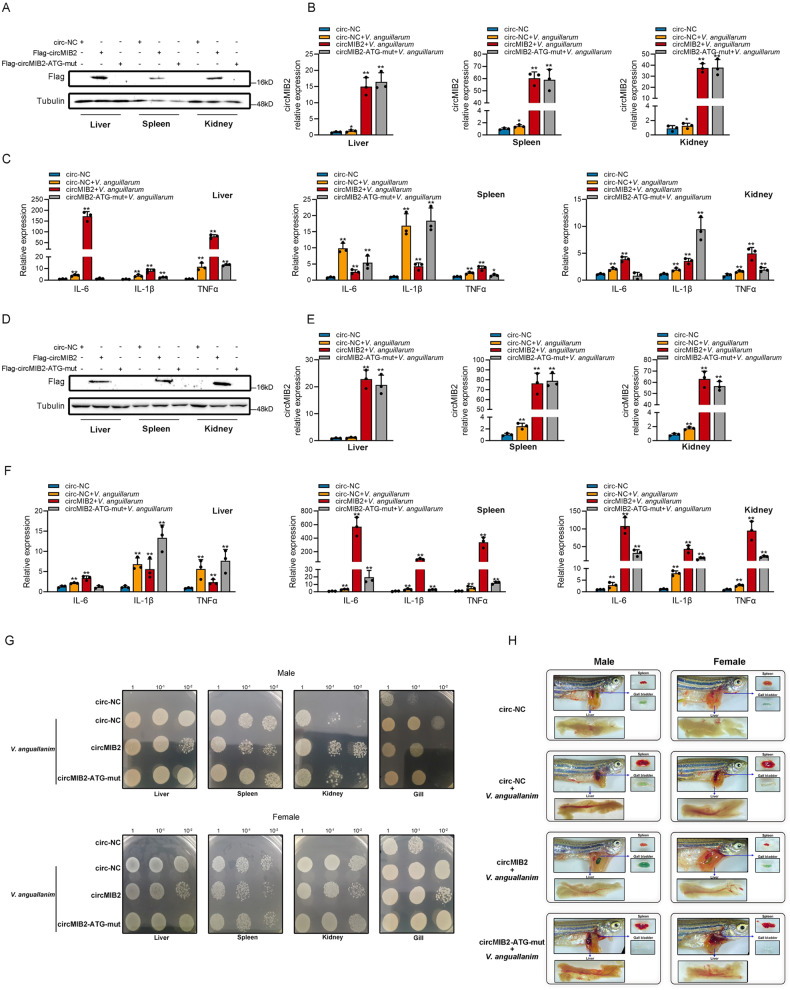
CircRNA therapy of circMIB2 promotes the innate immune response of zebrafish and inhibits the colonization of *V. anguillarum*. **A** Male zebrafish were intraperitoneally injected with circ-NC or Flag-circMIB2 or Flag-circMIB2-ATG-mut. WB was used to detect the expression of MIB2-134aa protein in the liver, spleen and kidney. Five zebrafish in each group. **B**, **C** Male zebrafish were intraperitoneally injected with circ-NC or Flag-circMIB2 or Flag-circMIB2-ATG-mut and then treated with *V. anguillarum*. The levels of circMIB2 in the liver, spleen, and kidney were detected by qPCR (**B**), and the levels of IL-6, IL-1β, and TNFα in the liver, spleen, and kidney were detected by qPCR (**C**). Three zebrafish in each group. **D** Perform the same treatment as (**A**) on the female zebrafish. **E** Perform the same treatment as (**B**) on the female zebrafish. **F** Perform the same treatment as (**C**) on the female zebrafish. **G** Male and female zebrafish were intraperitoneally injected with circ-NC or circMIB2 or circMIB2-ATG-mut and then treated with *V. anguillarum*. The number of *V. anguillarum* in the liver, spleen, and kidney was detected. Five zebrafish in each group. **H** Male and female zebrafish were intraperitoneally injected with circ-NC or circMIB2 or circMIB2-ATG-mut and then treated with *V. anguillarum*. *p < 0.05; **p < 0.01.

We want to know whether the role of circMIB2 in innate immune response has good versatility in other fish, so we chose the model organism zebrafish for the in vivo experiment. We injected the mixture of synthetic circRNA and Lipofectamine 3000 into the abdominal cavity of zebrafish and took the liver, spleen, kidney, and gill of zebrafish for subsequent experiments after 48–72 h of injection. We injected circ-NC, Flag-circMIB2, and Flag-circMIB2-ATG-mut into the abdominal cavity of zebrafish, respectively. After 48 h of injection, the liver, spleen, and kidney were taken, respectively, to detect the expression of MIB2-134aa protein. The experimental results showed that Flag-circMIB2 successfully expressed MIB2-134aa protein in the liver, spleen, and kidney of zebrafish male (Fig. 7A) and female fish (Fig. 7D), while circMIB2-ATG-mut did not express MIB2-134aa protein (Fig. 7A, D). We injected circ-NC, Flag-circMIB2, and Flag-circMIB2-ATG-mut into the abdominal cavity of zebrafish, respectively, and infected with *V. anguillarum* 24 h after injection. The experimental results showed that circMIB2 could increase the expression of circMIB2 in the liver of zebrafish male by about 20 times, the level of circMIB2 in the spleen by about 70 times, and the level of circMIB2 in the kidney by about 40 times (Fig. 7B). Similarly, in zebrafish females, the injection of circMIB2 can increase the level of circMIB2 in the liver by about 20 times, the level of circMIB2 in the spleen by about 90 times, and the level of circMIB2 in the kidney by about 70 times (Fig. 7E). In zebrafish males, circMIB2 can significantly promote IL-6, IL-1β, TNFα in liver and kidney under the condition of *V. anguillarum* infection (Fig. 7C). However, circMIB2 can’t promote the expression of IL-6 and IL-1β in spleen, and the promotion of TNFα is not very high (Fig. 7C). In zebrafish females, circMIB2 can significantly promote IL-6, IL-1β, TNFα in spleen and kidney under the condition of *V. anguillarum* infection (Fig. 7F). However, circMIB2 can’t promote the expression of IL-1β and TNFα in spleen, and the promotion of IL-6 is not very high (Fig. 7C). These results prove that the circRNA circMIB2 injected into the peritoneal cavity of zebrafish successfully entered the liver, spleen, and kidney cells of zebrafish, and can express MIB2-134aa protein, and promote the innate immune response of zebrafish. In male, circMIB2 could significantly inhibit the colonization of *V. anguillarum* on the liver, spleen and gill but could not inhibit the colonization of *V. anguillarum* on the kidney (Fig. 7G). In female, circMIB2 could significantly inhibit the colonization of *V. anguillarum* on the liver, spleen and kidney (Fig. 7G). Our results show that the organs of zebrafish infected with *V. anguillarum* have obvious changes. The liver will bleed and darken, the spleen will bleed and swell, and the gallbladder will turn from green to transparent (Fig. 7H). The zebrafish injected with circMIB2 can effectively resist the invasion of *V. anguillarum*. The results showed that compared with the control, the zebrafish injected with circMIB2 had normal liver color and only a small amount of bleeding, the gallbladder was normal green, and the spleen was not bleeding and swelling, while the zebrafish injected with circMIB2-ATG-mut had no such improvement (Fig. 7H). These results show that the circRNA therapy of circMIB2 can significantly inhibit the infection of *V. anguillarum* to zebrafish and promote the innate immune response of zebrafish.

## Discussion

Inexplicably, circRNAs do not possess the 5’-hat structure and 3’-poly (A) tail structure necessary for traditional translation. Through in-depth research, it was found that circRNAs have special translation methods, which are different from traditional translation methods. There are two types of circRNA translation methods that have been discovered, one of which is through internal ribosome entry site (IRES) element sequences that mediate translation [17]. For example, circZNF609, circMbl, and circFGFR1 induce the translation of small proteins through IRES element sequences and play corresponding functions [9, 18, 19]. Another one is through N6-methyladenosine (m^6^A) modification, and m6A-modified circRNAs can efficiently initiate translation, such as SHPRH-146aa can translate the small proteins through m^6^A modification [12]. In this study, we identified a novel circRNA, named circMIB2, whose host gene is E3 ubiquitin-protein ligase MIB2. Further studies showed that circMIB2 can encode a protein consisting of 134 amino acids and can participate in the regulation of the host’s innate immune response. It was found by prediction that the sequence of circMIB2 does not contain an IRES site, but there is an m6A modification site. Subsequently, we proved that circMIB2 can indeed mediate self-translation of proteins through m^6^A modification through RIP, MeRIP, and RNA pulldown experiments. Further studies show that METTL14 and METTL16 act as “writers” perform m^6^A modification on circMIB2, while ALKBH5 acts as “eraser” to demethylate circMIB2, and YTHDF1 and YTHDF3 act as “readers” to jointly promote the translation of proteins by circMIB2. These results all prove that circMIB2 is a circRNA capable of translating proteins and mediates the translation of proteins through m6A modification.

Although there are very few studies on the function of circRNA translation products, it can be determined that the translation products of these circRNAs often play a very important role [20–22]. According to the reported studies on circRNA-encoded proteins, we found that circRNA-encoded proteins mainly perform their functions in the following forms. The first form is not functionally related to host genes, and circRNA proteins can play some specific functions [23, 24], and the second form is related to the function of the host gene, such as indirectly regulating the stability or function of the host gene protein by binding to some proteins, SHPRH-146aa [12], AKT3-174aa [13], FBXW7-185aa [14], and β-catenin-370aa [16] all belong to this form; in addition, circRNA proteins can also affect their host gene proteins functions by directly interacting with host gene proteins, such as circLgr4-protein [15].

**Fig. 8:**
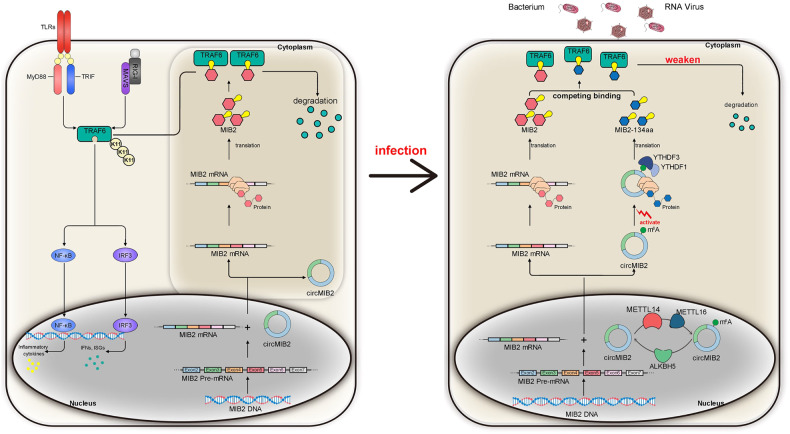
Schematic diagram of the mechanism MIB2-134aa competes with MIB2 for binding TRAF6 to regulate the ubiquitination. Under normal circumstances, circMIB2 does not translate to produce proteins. When SCRV virus or *V. anguillarum* infects the host, the pathway of circMIB2 translating proteins is activated. MIB2 could promote the TRAF6 protein degradation and repress TRAF6-mediated antiviral responses, thereby regulating viral replication and bacterial invasion. MIB2 promoted K11-linked ubiquitination of TRAF6, thereby inhibiting the antiviral responses and helping the virus escape. In addition, MIB2-134aa has the same domain as MIB2 protein. Through this domain, MIB2-134aa can compete with MIB2 for binding to the same position of TRAF6, thereby inhibiting the ubiquitination of TRAF6 and promoting the host’s innate immune response.

To develop a better mechanistic understanding of the circRNA proteins-host gene proteins relationship and the significance of circRNA-encoded proteins, functional studies based on such circRNA proteins with the same domain as their host gene proteins are necessary. The following hypothesis is put forward regarding the relationship between such circRNA proteins and their host genes: such circRNA proteins can perform the same functions as host gene proteins through the same domain to compensate or competitively regulate the function of host genes. As we expected, our results confirm that circRNA-encoded proteins can compete with host gene proteins to regulate the host’s innate immune response (Fig. 8). We believe that in normal circumstances, fish infected with SCRV or *V. anguillarum* typically experience two outcomes. First, the fish may succumb to the infection, resulting in SCRV or *V. anguillarum* prevailing. Alternatively, the fish may survive, with SCRV or *V. anguillarum* being eliminated or coexisting with the host. Regardless of the outcome, there is an ongoing arms race between the host and the pathogen. We found that MIB2 benefits the pathogen, while circMIB2 serves as a means for the host to counteract the hijacking of MIB2. The lower level of circMIB2 suggests that, at this stage, the advantage lies with the pathogen rather than the host. However, the host has implemented a fast and effective strategy to block the pathogen from hijacking its genes. Specifically, circMIB2 promotes the level of TRAF6 by producing the MIB2-134aa protein. This protein competes with MIB2 for the binding site of TRAF6, effectively preventing MIB2 from degrading TRAF6. Therefore, the role of MIB2-134aa is to alleviate the degradation of TRAF6 by MIB2. However, at this stage, due to the disparity in quantity between the two entities, MIB2-134aa cannot completely prevent TRAF6 degradation; it can only partially delay the process initiated by MIB2. The significance of our study lies in the discovery of a critical target that is hijacked by pathogens. Even if the level of circMIB2 in the host is low, it is not a concern because we have demonstrated that artificially increasing the levels of circMIB2 or MIB2-134aa protein effectively alleviates the infection caused by SCRV or *V. anguillarum* in the host. This finding opens up new possibilities for combating infections by providing a means to counteract the pathogen-driven hijacking of host genes.

In addition, through the circRNA therapy of circMIB2 in zebrafish, it is not difficult to find that the MIB2-134aa protein encoded by circMIB2 can promote the innate immune response of the host, and can significantly inhibit the infection of *V. anguillarum*, which undoubtedly strongly proves the effectiveness of circRNA therapy. In addition, compared with mRNA therapy, circRNA therapy has the advantages of low immunogenicity, high stability, and long duration of curative effect. Therefore, circRNA therapy is being extensively studied in human medicine, but it has not started in other species. Our research provides a reference for the first time for the application of circRNA therapy to the treatment of pathogenic diseases in economic fish.

## Methods

### Sample and challenge

Miiuy croaker, *Miichthys miiuy* (∼50 g), was obtained from Zhoushan Fisheries Research Institute, Zhejiang Province, China. Fish was acclimated in aerated seawater tanks at 25 °C for 6 weeks before experiments. Experimental procedures for SCRV infection were performed as described [25].

### Cell culture and treatment

MKC, MSpC, MIC, MBrC, MMC and MLC cells were cultured in L-15 medium (HyClone) supplemented with 15% fetal bovine serum (FBS; Gibco), 100 U/ml penicillin, and 100 μg/ml streptomycin at 26 °C [25]. Epithelioma papulosum cyprini cells (EPC) were maintained in medium 199 medium (Invitrogen) supplemented with 10% FBS, 100 U/ml penicillin, and 100 mg/ml streptomycin at 28 °C in 5% CO_2_ [26].

### Plasmids construction

To construct circMIB2 plasmid (circMIB2-P) and Flag-circMIB2 plasmid (Flag-circMIB2-P), the full-length circMIB2 sequence or full-length circMIB2 sequence with Flag sequence was amplified by specific primer pairs and cloned into pLC5-circ vector (Geneseed Biotech), which contained a front and back circular frame to promote RNA circularization. Construction of initial codon mutant of Flag-circMIB2-P plasmid (Flag-circMIB2-ATG-mut-P). To construct the Flag-circMIB2-P plasmid with deleted back circular frame, which promotes RNA circularization (Linear-AG-MIB2-FL-P), the back circular frame was deleted on the basis of the Flag-circMIB2-P plasmid. To construct a plasmid Linear-Flag-MIB2-134aa-P, the complete circMIB2 coding region sequence and Flag sequence were amplified and inserted into pcDNA3.1 vector (Invitrogen). To construct the m^6^A motif mutant of Flag-circMIB2-P plasmid on the basis of Flag-circMIB2-P plasmid. The STING, MAVS, TRIF, and TRAF6 plasmids were constructed as described [25–27]. To construct m6A-related genes plasmid, the full-length genes sequence with Flag sequence was amplified by specific primer pairs and cloned into pcDNA3.1 vector (Invitrogen).

### Prediction of the secondary structures of RNAs by UNAfold and circularization in vitro

The secondary structures of circMIB2 were predicted at [NaCl] = 1.0 M and 37 °C by the UNAfold Web Server. According to the prediction results, the optimal cyclization site of circMIB2 was determined, and the complete linear RNA of circMIB2 (L-circMIB2) was synthesized from this cyclization site. For ligation experiments, a phosphate was in advance introduced to the 5’-position of L-circMIB2 by using T4 polynucleotide kinase (Beyotime) and T4 RNA Ligase 2 (Beyotime) [28]. Finally, circMIB2 was purified by ethanol precipitation.

### RNA oligoribonucleotides

The RNA interferences for circMIB2 are as follows: si-circMIB2-1 sequence was 5’-CACACUCCCAGCCUCACCCTT-3’. And the si-circMIB2-2 sequence was 5’-CUCCCAGCCUCACCCCUGATT-3; The scrambled control RNA sequences were 5’- GGCACACUCCCAGCCAACAAATT-3’. The RNA interference for MIB2 is as follows: si-MIB2 sequence was 5’- UGUUACAUGAACAACAAGCTT-3’. The scrambled control RNA sequences were 5’- UUCUCCGAACGUGUCACGUTT-3’.

### Cell transfection

Transient transfection of cells with siRNA was performed in 24-well plates using Lipofectamine RNAiMAX (Invitrogen), and cells were transfected with DNA plasmids or synthetic circRNA were performed using Lipofectamine 3000 (Invitrogen) according to the manufacturer’s instructions. For functional analyses, the plasmid (500 ng per well) or control vector (500 ng per well), the circRNA (500 ng per well) or circ-NC (500 ng per well), and siRNA (100 nM) were transfected into cells in culture medium and then harvested for further detection.

### Zebrafish injection and infection

Zebrafish, *Danio rerio* (4 months old) were maintained at 28 °C in tanks. Mix the synthesized circRNA and Lipofectamine 3000 (Invitrogen), and then inject the mixture into the abdominal cavity of zebrafish (10 μg). After 24 h of injection of circRNA, the experiment of *V. anguillarum* infection was started. The zebrafish in the control group was injected with 20 μl of normal saline intraperitoneally, and the zebrafish in the infection group was injected with 20 μl of *V. anguillarum* (1 × 10^8^ CFU/ml).

### Bacterial colonization

Add the liver, spleen, kidney or gill of the above zebrafish into sterile centrifuge tubes, add sterile physiological saline and steel ball to shake and break. Then the broken tissue solution is coated on the plate, and the plate is incubated at 37 °C for 12 h.

### RNA extract and quantitative real-time PCR

For the isolation and purification of both cytoplasmic and nuclear RNA from MSpC and MIC cells, the Cytoplasmic & Nuclear RNA Purification Kit has been used according to the manufacturer’s instructions (Norgen Biotek). The total RNA of tissue or cell samples was isolated with TRIzol Reagent (Invitrogen), and the cDNA was synthesized using the FastQuant RT Kit (Tiangen). The expression patterns of each gene were performed by using SYBR Premix Ex TaqTM (Takara). GAPDH was employed as endogenous controls for mRNA, respectively. Primer sequences are displayed in Supplementary Table 1.

### Luciferase report assay

To determine the functional regulation of MIB2-134aa, cells were co-transfected the plasmid of STING, MAVS, TRIF, or TRAF6 and circMIB2 or circMIB2-ATG-mut, together with IRF3, ISRE, NF-κB, and IL-1β luciferase reporter gene plasmids, phRL-TK *Renilla* luciferase plasmid, negative controls. At 48 h post-transfection, the cells were lysed for reporter activity using the dual-luciferase reporter assay system (Promega). All the luciferase activity values were achieved against the *renilla* luciferase control. Ratios of *renilla* luciferase readings to firefly luciferase readings were taken for each experiment, and triplicates were averaged.

### Antibody generation and Western Blotting

A polyclonal antibody against the MIB2-134aa protein produced by circMIB2 was obtained by inoculating rabbits (GenScript). Cellular and tissue lysates were generated by using 1 × SDS-PAGE loading buffer. Proteins were extracted from cells and measured with the BCA Protein Assay kit (Vazyme), then subjected to SDS-PAGE (8%) gel and transferred to PVDF (Millipore) membranes by semidry blotting (Bio-Rad Trans Blot Turbo System). The antibody against TRAF6 was diluted at 1:500 (Abcam); The antibody against MIB2 was diluted at 1:500 (Abcam); The antibody against MIB2-134aa was diluted at 1:200 (GenScript); anti-Flag, anti-HA, anti-Myc, and anti-Tubulin monoclonal antibody were diluted at 1:2000 (Sigma); and HRP-conjugated anti-rabbit IgG or anti-mouse IgG (Abbkine) at 1:5000. The immunoreactive proteins were detected by using WesternBright^TM^ ECL (Advansta). The digital imaging was performed with a cold CCD camera.

### Immunoprecipitation assay

For immunoprecipitation (IP) experiments, MSpC cells, EPC cells were seeded onto a 10-cm^2^ plate overnight and then were co-transfected with 5 μg indicated plasmids, and the specific steps were performed as described [29].

### MeRIP-qPCR assays

Total RNA was isolated using TRIzol reagent. Then the cleaved RNA fragments were incubated for 2 h at 4 °C with m^6^A-specific antibody (Germany) in IP buffer. Then the IP RNA was reverse-transcribed to create the cDNA by SuperScript™ II Reverse Transcriptase (Invitrogen). RNA was extracted from the remaining beads, and qPCR was used to evaluate the expression levels of circMIB2.

### RNA immunoprecipitation assay (RIP)

RIP experiments were performed by using the Magna RIP RNA-Binding Protein Immunoprecipitation Kit (Millipore) following the manufacturer’s protocol. The RIP assay was also conducted in MSpC cells (~2.0 × 10^7^) transfected with YTHDF1 and YTHDF3. After 48 h transfection, the MSpC cells were used in RIP assays via the Magna RIP^TM^ RNA-Binding Protein Immunoprecipitation Kit (Millipore) and an anti-flag antibody following the manufacturer’s protocol.

### RNA pulldown assay

We first synthesized the circRNA of the m^6^A mutant of circMIB2 (MS2-circMIB2-m^6^A-mut) and circMIB2 (MS2-circMIB2) with MS2 sequence. The MS2-RNA pulldown assay was conducted in MSpC cells transfected with YTHDF1, YTHDF3, pMS2-GFP (Addgene) plasmids and MS2-circMIB2, MS2-circMIB2-m^6^A-mut circRNA, and the specific steps were performed as described [30].

### LC-MS analysis

CircMIB2-134aa proteins were separated on SDS-PAGE gels, and bands were manually excised from the gel and digested with sequencing-grade trypsin (Promega). The digested proteins were analyzed using a QExactive mass spectrometer (Thermo). Fragment spectra were analyzed using the National Center for Biotechnology Information nonredundant protein database with Mascot (Matrix Science) [31].

### RNA FISH

The RNA FISH assay was performed using Cy3-labeled circMIB2 probe (5’-Cy3-AACAGATCAGGGGTGAGGCTGGGAGTGTG-3’) and FAM-labeled MIB2 probe (5’-Cy3-CATCTCCTCGGTCAGTGATTCTA-3’). Subsequently, the Fluorescent In Situ Hybridization test kit (GENESEED) was used for detection in MSpC and MIC cells.

### Statistical analysis

Data are expressed as the mean ± SE from at least three independent triplicated experiments. Student’s t-test was used to evaluate the data. The relative gene expression data was acquired using the 2^−^^∆∆CT^ method, and comparisons between groups were analyzed by one-way analysis of variance (ANOVA) followed by Duncan’s multiple comparison tests [32]. A value of *p* < 0.05 was considered significant.

## Supplementary information

Supplementary Figure Legends

Supplementary Figure 1

Supplementary Figure 2

Supplementary Table 1

Original Data File-1

Original Data File-2

## Data Availability

The data that support the findings of this study are available in the “Methods” and/or supplementary material of this article.
