# Supplementary material for: CircMIB2 therapy can effectively treat pathogenic infection by encoding a novel protein
Source: Cell Death Dis. 2023 Aug 31;14(8):578. doi: 10.1038/s41419-023-06105-3 (PMC10471593; doi:10.1038/s41419-023-06105-3)
Supplement: Supplementary file 1 — Supplementary Figure Legends [file 41419_2023_6105_MOESM1_ESM.docx]

**Supplemental Fig. 1 Expression profiles and characterization of circMIB2.** (A) Relative expression of MIB2 and circMIB2 in indicated cell lines was determined by qPCR, including RNase R treated group and not treated group. (B) PCR validated the existence of circMIB2 in MSpC and MIC cell lines. circMIB2 was amplified by divergent primers in cDNA but not gDNA. GAPDH was used as a negative control. All data represent the means ± SE from three independent triplicate experiments. *, *p*<0.05; **, *p*<0.01.

**Supplemental Fig. 2 Detection of circMIB2.** (A) After transfecting the circMIB2-P plasmid into MIC cells, the levels of MIB2-134aa protein were detected; after transfecting si-circMIB2-1 into MSpC cells, the levels of MIB2-134aa protein were detected. (B) Immunoprecipitation and immunoblot analysis of MIB2-134aa in MSpC cells. The differential gel bands were extracted and subjected to LC-MS. (C) Protein levels of ISG15 in MSpC cells after transfected with si-NC or si-circMIB2-1 or circ-NC or circMIB2 after SCRV infection, and the protein levels of TNFα and IL-8 in MSpC cells after transfected with si-NC or si-circMIB2-1 or circ-NC or circMIB2 after *V. anguillarum* infection. (D) Protein levels of ISG15 in MSpC cells after transfected with si-NC or si-MIB2 or vector or MIB2 plasmid after SCRV infection, and the protein levels of TNFα and IL-8 in MSpC cells after transfected with si-NC or si-MIB2 or vector or MIB2 plasmid after *V. anguillarum* infection. All data represent the means ± SE from three independent triplicate experiments. *, *p*<0.05; **, *p*<0.01.
