# Supplementary figures and images for: CircMIB2 therapy can effectively treat pathogenic infection by encoding a novel protein
Source: Cell Death Dis. 2023 Aug 31;14(8):578. doi: 10.1038/s41419-023-06105-3 (PMC10471593; doi:10.1038/s41419-023-06105-3)

# Supplementary Figure 1

A

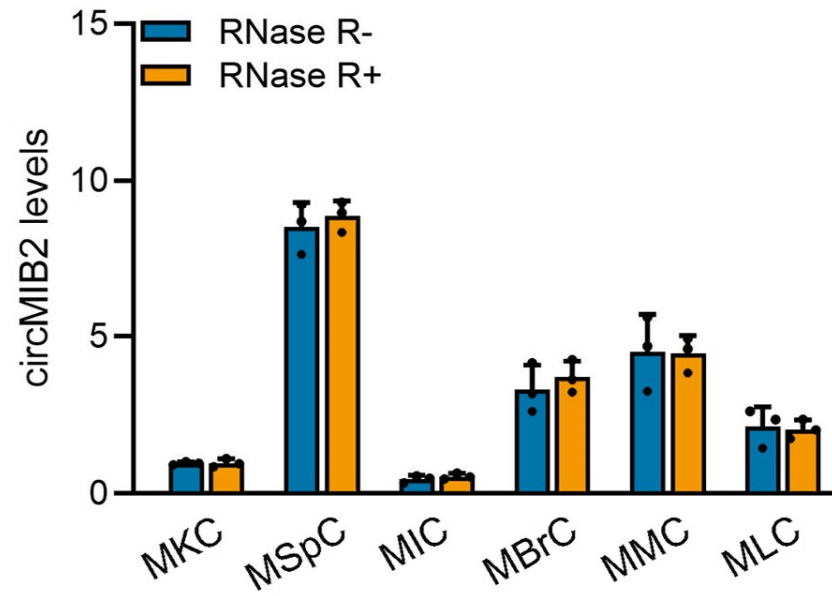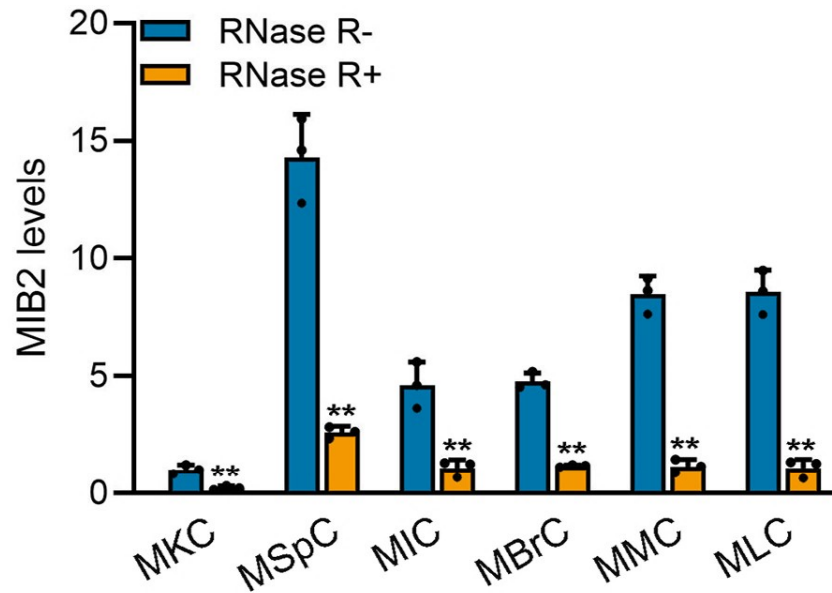

B

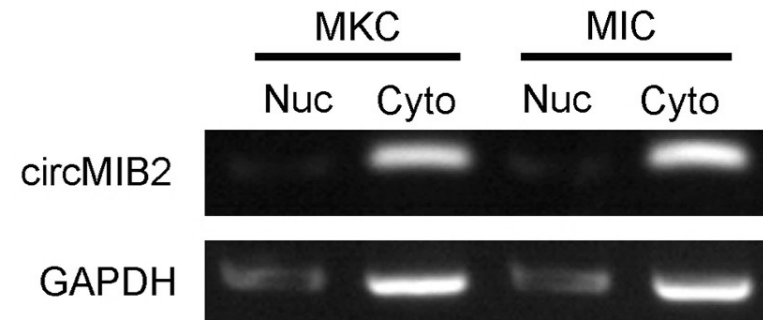

Supplement: Supplementary file 2 — Supplementary Figure 1 [file 41419_2023_6105_MOESM2_ESM.pdf]

# Supplementary Figure 2

A

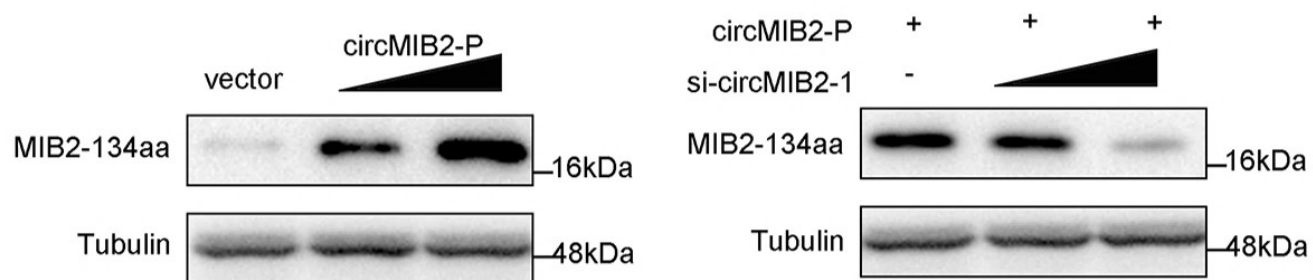

B

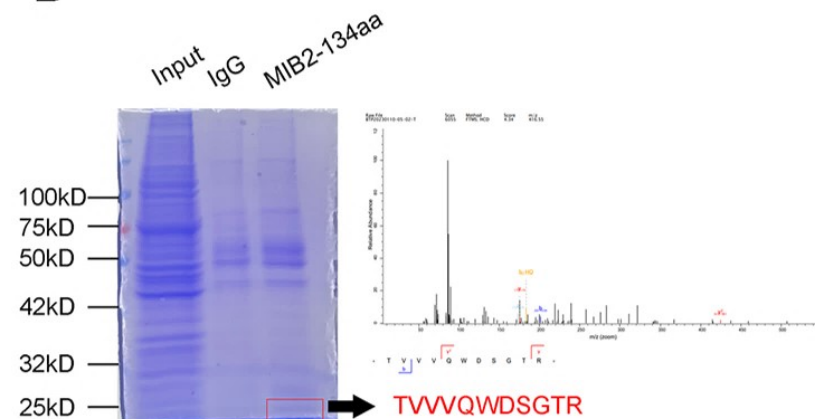

C

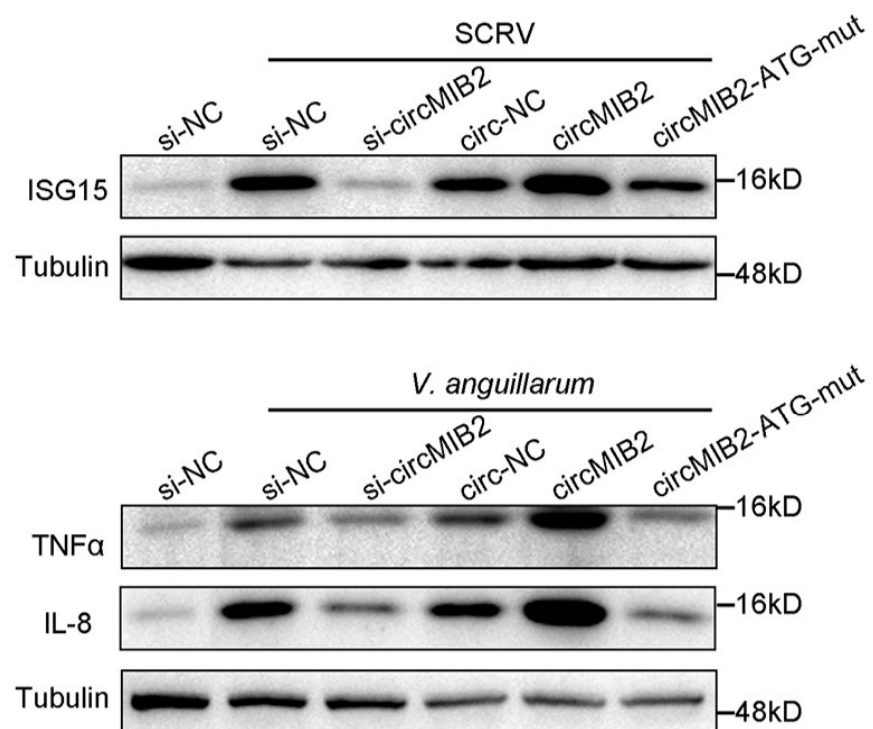

D

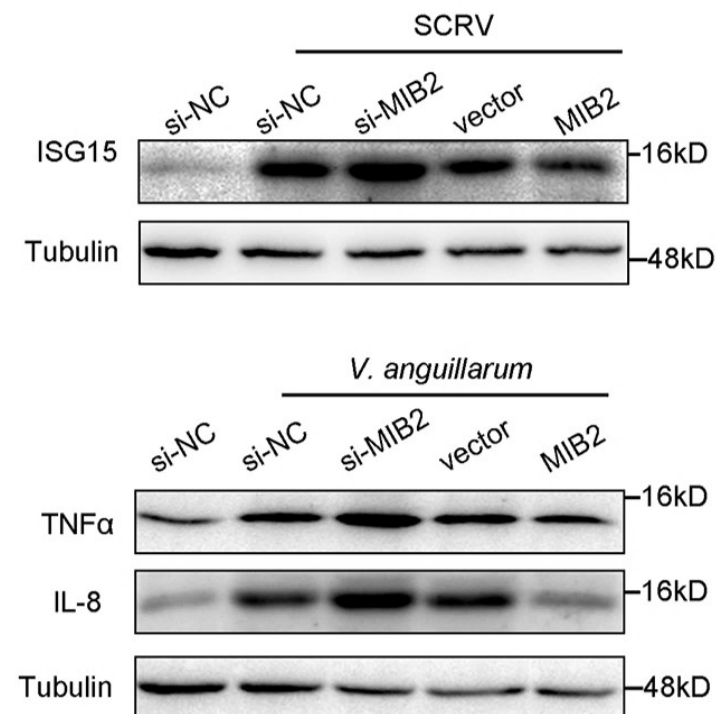

Supplement: Supplementary file 3 — Supplementary Figure 2 [file 41419_2023_6105_MOESM3_ESM.pdf]

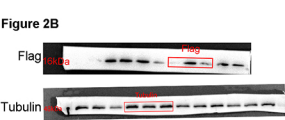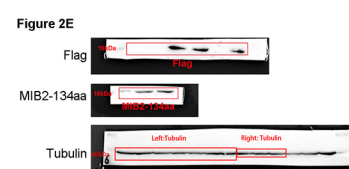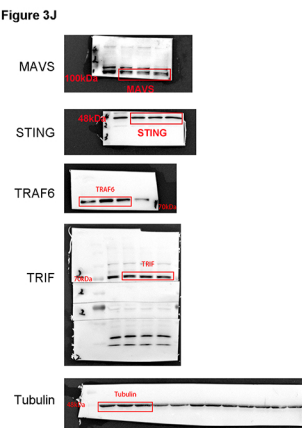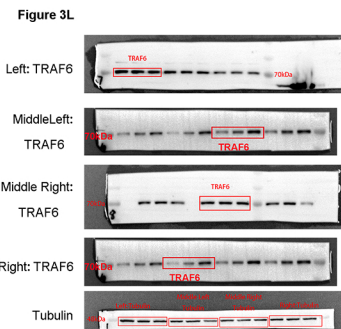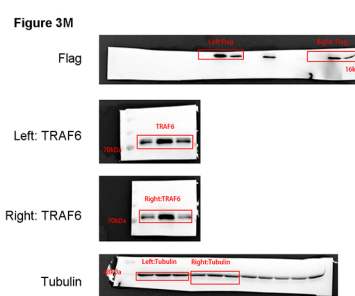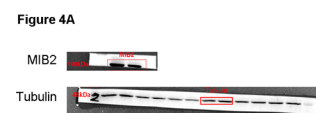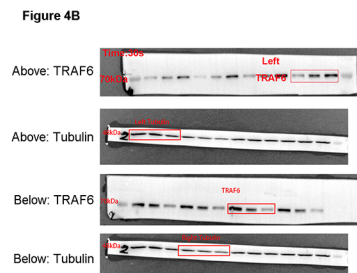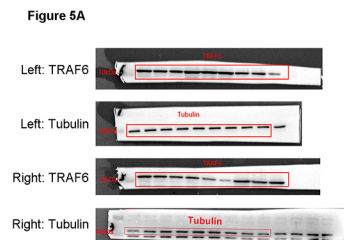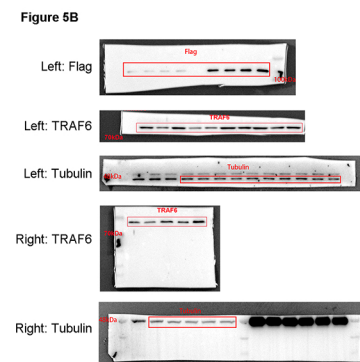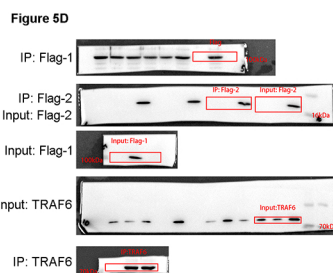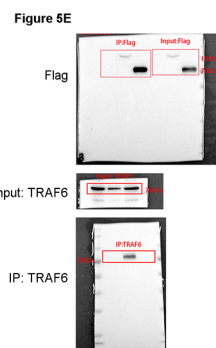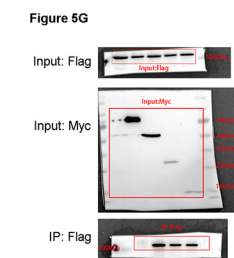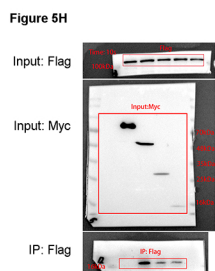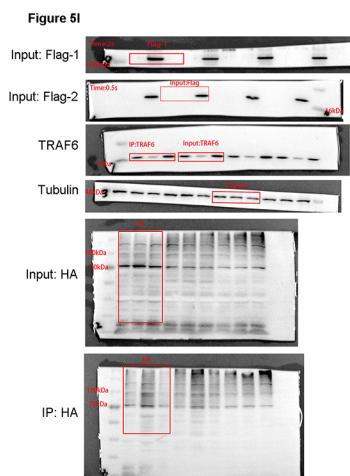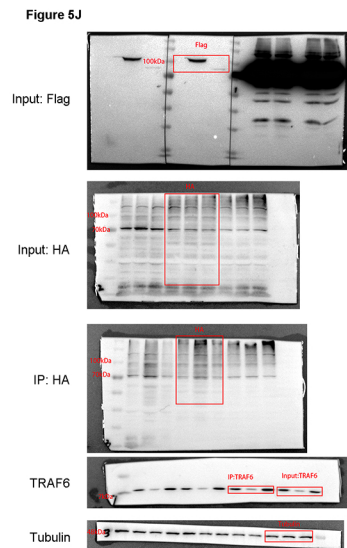

Supplement: Supplementary file 5 — Original Data File-1 [file 41419_2023_6105_MOESM5_ESM.pdf]
