# Supplementary material for: CircMIB2 therapy can effectively treat pathogenic infection by encoding a novel protein
Source: Cell Death Dis. 2023 Aug 31;14(8):578. doi: 10.1038/s41419-023-06105-3 (PMC10471593; doi:10.1038/s41419-023-06105-3)
Supplement: Supplementary file 4 — Supplementary Table 1 [file 41419_2023_6105_MOESM4_ESM.docx]

**Supplementary Table 1** PCR primer information in this study.

| Primer | Sequences (5’-3’) |
| --- | --- |
| TRAF6-qRT-F | ATGATGGAAAAGGAACGGGAAT |
| TRAF6-qRT-R | TCGGACAGCGAACAGTTAGTGA |
| TNF-α-qRT-F | GTTTGCTTGGTACTGGAATGG |
| TNF-α-qRT-R | TGTGGGATGATGATCTGGTTG |
| IL-8-qRT-F | AGCAGCAGAGTCTTCGT |
| IL-8-qRT-R | TCTTCGCAGTGGGAGTT |
| IL-1β-qRT-R | CATAAGGATGGGGACAACGAG |
| IL-1β-qRT-R | TAGGGGACGGACACAAGGGTA |
| MX1-qRT-F | GCTGCTTGTTTACTCCCA |
| MX1-qRT-R | ACCTGCATCATCTCCCTC |
| ISG15-qRT-F | TGAACGGACAGAAGACGC |
| ISG15-qRT-R | TGAGGAATACCTGCATGG |
| Viperin-qRT-F | ACCCGTCCAAGTCCATAC |
| Viperin-qRT-R | TCATGTCAGCTTTGCTCC |
| SCRV-qRT-F | GGGCTGGATGATAGACGATTG |
| SCRV-qRT-R | TGGCGGAGGTGCTTGATATGG |
| GAPDH-qRT-F | ACCTTCACTCCTCCATCTT |
| GAPDH-qRT-R | AGGTCACAGACACGGTTG |
| GAPDH-divergent-F | CTGAAAGTAAACCAAACA |
| GAPDH-divergent-R | GTTTGCTCTCTGTGGGACT |
| GAPDH-convergent-F | AGTAAACCAAACAAGCCA |
| GAPDH-convergent-R | TTCAGCACCACAGACAGA |
| circMIB2-divergent-F | ACGGCACACTCCCAGCCTCAC |
| circMIB2-divergent-R | CCTGGCAGCACTGTCAATCCC |
| circMIB2-convergent-F | GCATGCGCGTGGTGCGTG |
| circMIB2-convergent-R | CCAATTTGAGCGTTGTCA |
| circMIB2-m6A-qRT-F | CCGGAAACATCCCTGG |
| circMIB2-m6A-qRT-R | TGCTGCCTCTGCTGGG |
| circMIB2-F | AATATTTCTTCTTTCGAATTCTAATACTTTCAGTCACCCCTGATCTGTTCCCG |
| circMIB2-R | TGGAGTTGTTAGCTAGGATCCAGTTGTTCTTACGGCTGGGAGTGTGCCGTC |
| circMIB2-Flag-F | GAATTCTAATACTTTCAGTCACCCCGATGACGACAAGTGATCTGTTCCCG |
| circMIB2-Flag-R | GGATCCAGTTGTTCTTACGTCTTTGTAGTCGGCTGGGAGTGTGCCGTC |
| circMIB2-ATG-mut-F | AGAGGCAGCAAAGAGGTGGGCATGCGCGTGGT |
| circMIB2- ATG-mut-R | ACCTCTTTGCTGCCTCTGCTGGGGCCTGGCCT |
| circMIB2-T7-F | TAATACGACTCACTATAGGGGGTGCCGTCCCTAACCACA |
| circMIB2-T7-R | AGGGATGTTTCCGGGAACAGATCAGGGGTGAGGCTGGGAGTGTGCCGTCT |
| circMIB2-Flag-m6A-1F | GATTGCCAGTGCTGCCAGGCCCCGGCCGTTGC |
| circMIB2-Flag-m6A-1R | TGGCAGCACTGGCAATCCCGCTGCTGCTGCGC |
| circMIB2-Flag-MS2-F | ACATGAGGATCACCCATGTCTGCAGTCTGTTCCCGGAAACATCCC |
| circMIB2-Flag-MS2-R | CATGGGTGATCCTCATGTTTTCTAGTCACTTGTCATCGTCGTCCTTG |
| MIB2-qRT-1F | GAGAACTCGGAGCAGC |
| MIB2-qRT-1R | TCAGACGAATAACTGG |
| MIB2-170aa-Flag-1F | CGGAATTCATGGAGGTGGGCATGCGC |
| MIB2-170aa-Flag-1R | CGGGATCCTCACTTGTCGTCATCGTCTTTGTAGTCGGGGTGAGGCTGGGAGTGTGCCGTC |
| TRAF6-1F | CGCGGATCCATGGCTTGCATTGACAGC |
| TRAF6-1R | CCGGAATTCTTTCCCCATTCTGGTGTC |
| TRAF6-(140-597aa)-1F | CGCGGATCCACTAACTGTTCGCTGTCCG |
| TRAF6-(140-597aa)-1R | CCGGAATTCTTTCCCCATTCTGGTGTC |
| TRAF6-(280-597aa)-1F | CGCGGATCCAATGCGCTACATGGCGGAG |
| TRAF6-(280-597aa)-1R | CCGGAATTCTTTCCCCATTCTGGTGTC |
| TRAF6-(400-597aa)-1F | CGCGGATCCACACGGTATCTTCATCTGG |
| TRAF6-(400-597aa)-1R | CCGGAATTCTTTCCCCATTCTGGTGTC |
| METTL3-Flag-1F | CCCAAGCTTCTCGTCATGTCGGACACAT |
| METTL3-Flag-1R | CCGGAATTCTGCGGGGATCACATACAG |
| METTL14-Flag-1F | CCCAAGCTTACGAAGGAGAAAATGAACAGTC |
| METTL14-Flag-1R | CGCGGATCCTGTGATTGGAGATTCATAAGGC |
| METTL16-Flag-1F | CCCAAGCTTAACAAGGAAGACTCCAACA |
| METTL16-Flag-1R | CCGGAATTCCTTCTCAAAAGCTGTATGC |
| FTO-Flag-1F | CGGGGTACCCACAACTCCAGGAACATG |
| FTO-Flag-1R | TGCTCTAGAGCCTCTACATTAAGAAAAGC |
| ALKBH5-Flag-1F | CCCAAGCTTGGCTATCTGTCAGCTACTAC |
| ALKBH5-Flag-1R | CCGGAATTCCTTCGTCTGCCAGAAAC |
| YTHDF1-Flag-1F | CGGGGTACCCATTTCAACATGACCACCAA |
| YTHDF1-Flag-1R | CCGGAATTCGCAGCCGTCTTCTGTTTACT |
| YTHDF3-Flag-1F | CCCAAGCTTTCAGTGCAAAACGGATCAAT |
| YTHDF3-Flag-1R | CGCGGATCCGCCTTTCCTCCTTTGTGGTT |
| EIF3a-Flag-1F | GACGACAAGAAGCTTGGTACCATGCCGGCGTATTTTCAACG |
| EIF3a-Flag-1R | GTTCACGGACTGTCTTCTTCTTGATCTGCTCGTG |
| EIF3a-Flag-2F | GAAGAAGACAGTCCGTGAACGGCTGGAGC |
| EIF3a-Flag-2R | TGATGGATATCTGCAGAATTCTCAGCGGCGGACAGTGGT |
| EIF4G2-Flag-1F | GACGATGACGACAAGAAGCTTATGCTGGGCAACATCAAATTC |
| EIF4G2-Flag-1R | TGATGGATATCTGCAGAATTCTCAGAAATCTTCACCCTCTGACTCC |
| MIB2-F | GACGATGACGACAAGAAGCTTATGGAGGTGGGCATGCGC |
| MIB2-R | TGATGGATATCTGCAGAATTCTCAGACGAATAACTGGATCCGC |
| MIB2-△(1-132aa)-F | GACGATGACGACAAGAAGCTTGTGAGCCTGGCACCGAGG |
| MIB2-△(1-132aa)-R | TGATGGATATCTGCAGAATTCTCAGACGAATAACTGGATCCGC |
