# Supplementary material for: CircMIB2 therapy can effectively treat pathogenic infection by encoding a novel protein
Source: Cell Death Dis. 2023 Aug 31;14(8):578. doi: 10.1038/s41419-023-06105-3 (PMC10471593; doi:10.1038/s41419-023-06105-3)

**Figure 5K**

Input: Flag

Input: HA

Input: TRAF6

Input: Tubulin

IP: HA

IP: TRAF6

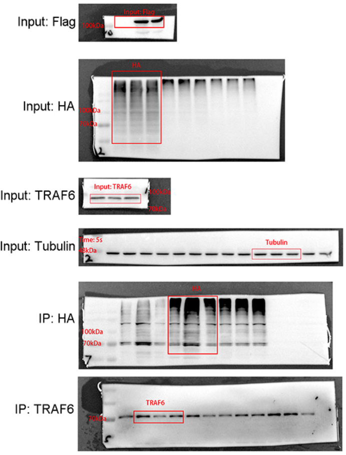

**Figure 5L**

Input: Flag-MIB2

Input: Flag-MIB2-134aa

Tubulin

IP: Flag-MIB2

IP: Flag-MIB2-134aa

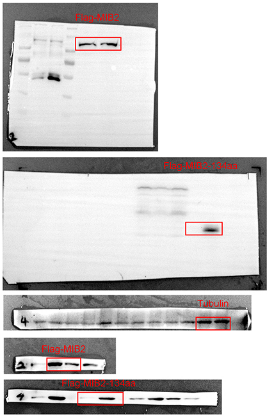

**Figure 5M**

Input: Flag-1

Input: Flag-2

Input: HA

Input: TRAF6

Input: Tubulin

IP: HA

IP: TRAF6

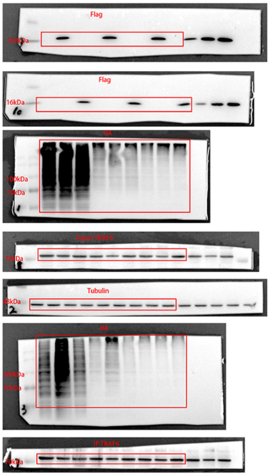

**Figure 6A**

Flag-circMIB2

Myc

Tubulin

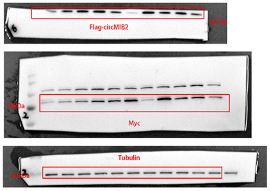

**Figure 6B**

Flag

Myc

Tubulin

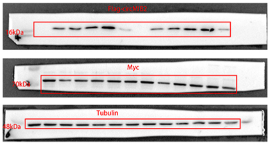

**Figure 6C**

Flag

Myc

Tubulin

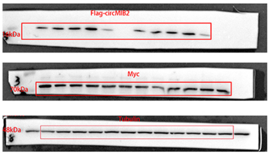

**Figure 6D**

Flag

Myc

Tubulin

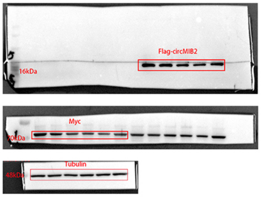

**Figure 6H**

Left: GFP
Right: IP-GFP

Left: Input-Flag

Left: IP-Flag

Left: Tubulin

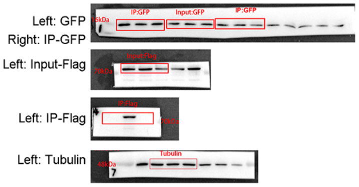

Right: Input-Flag

Right: Input-GFP

Right: Tubulin

Right: IP-Flag

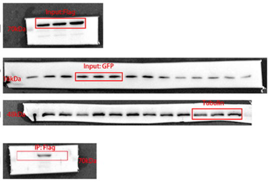

**Figure 7A**

Flag

Tubulin

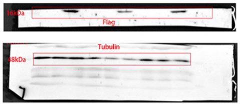

**Figure 7D**

Flag

Tubulin

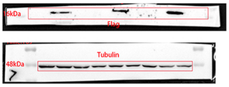

**Figure S2A**

Left: MIB2-134aa

Left: Tubulin

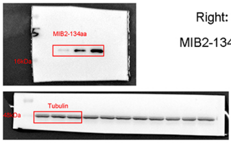

Right: MIB2-134aa

Right: Tubulin

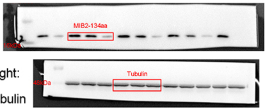

**Figure S2C**

Above: ISG15

Above: Tubulin

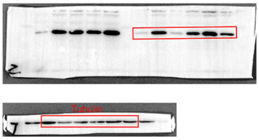

Below: IL-8

Below: TNFα

Below: Tubulin

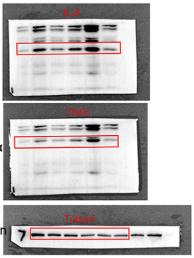

**Figure S2D**

Above: ISG15

Above: Tubulin

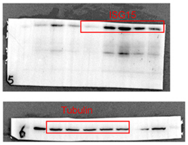

Below: IL-8

Below: TNFα

Below: Tubulin

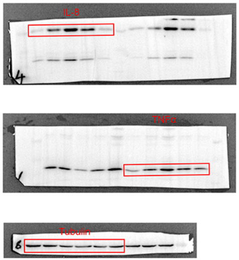

Supplement: Supplementary file 6 — Original Data File-2 [file 41419_2023_6105_MOESM6_ESM.pdf]
